# What Do We Have to Know about PD-L1 Expression in Prostate Cancer? A Systematic Literature Review (Part 6): Correlation of PD-L1 Expression with the Status of Mismatch Repair System, *BRCA*, *PTEN*, and Other Genes

**DOI:** 10.3390/biomedicines10020236

**Published:** 2022-01-22

**Authors:** Andrea Palicelli, Stefania Croci, Alessandra Bisagni, Eleonora Zanetti, Dario De Biase, Beatrice Melli, Francesca Sanguedolce, Moira Ragazzi, Magda Zanelli, Alcides Chaux, Sofia Cañete-Portillo, Maria Paola Bonasoni, Stefano Ascani, Antonio De Leo, Guido Giordano, Matteo Landriscina, Giuseppe Carrieri, Luigi Cormio, Jatin Gandhi, Davide Nicoli, Enrico Farnetti, Simonetta Piana, Alessandro Tafuni, Martina Bonacini

**Affiliations:** 1 Pathology Unit, Azienda USL-IRCCS di Reggio Emilia, 42123 Reggio Emilia, Italy; Alessandra.Bisagni@ausl.re.it (A.B.); Eleonora.Zanetti@ausl.re.it (E.Z.); Moira.Ragazzi@ausl.re.it (M.R.); Magda.Zanelli@ausl.re.it (M.Z.); mariapaola.bonasoni@ausl.re.it (M.P.B.); simonetta.piana@ausl.re.it (S.P.); alessandro.tafuni@unipr.it (A.T.); 2 Clinical Immunology, Allergy and Advanced Biotechnologies Unit, Azienda USL-IRCCS di Reggio Emilia, 42123 Reggio Emilia, Italy; Stefania.Croci@ausl.re.it (S.C.); Martina.Bonacini@ausl.re.it (M.B.); 3 Department of Pharmacy and Biotechnology (FABIT), University of Bologna, 40126 Bologna, Italy; dario.debiase@unibo.it; 4 Fertility Center, Department of Obstetrics and Gynecology, Azienda USL-IRCCS di Reggio Emilia, 42123 Reggio Emilia, Italy; Beatrice.Melli@ausl.re.it; 5 Clinical and Experimental Medicine PhD Program, University of Modena and Reggio Emilia, 41121 Modena, Italy; 6 Pathology Unit, Policlinico Riuniti, University of Foggia, 71122 Foggia, Italy; francesca.sanguedolce@unifg.it; 7 Department of Scientific Research, School of Postgraduate Studies, Norte University, Asuncion 1614, Paraguay; alcideschaux@uninorte.edu.py; 8 Department of Pathology, University of Alabama at Birmingham, Birmingham, AL 35294, USA; scaneteportillo@uabmc.edu; 9 Pathology Unit, Azienda Ospedaliera Santa Maria di Terni, University of Perugia, 05100 Terni, Italy; s.ascani@aospterni.it; 10 Haematopathology Unit, CREO, Azienda Ospedaliera di Perugia, University of Perugia, 06129 Perugia, Italy; 11 Molecular Diagnostic Unit, Azienda USL Bologna, Department of Experimental, Diagnostic and Specialty Medicine, University of Bologna, 40138 Bologna, Italy; antonio.deleo@unibo.it; 12 Medical Oncology Unit, Department of Medical and Surgical Sciences, University of Foggia, 71122 Foggia, Italy; guido.giordano@unifg.it (G.G.); matteo.landriscina@unifg.it (M.L.); 13 Department of Urology and Renal Transplantation, University of Foggia, 71122 Foggia, Italy; giuseppe.carrieri@unifg.it (G.C.); luigi.cormio@unifg.it (L.C.); 14 Department of Pathology and Laboratory Medicine, University of Washington, Seattle, WA 98195, USA; jgandhi@uw.edu; 15 Molecular Biology Laboratory, Azienda USL-IRCCS di Reggio Emilia, 42123 Reggio Emilia, Italy; Davide.Nicoli@ausl.re.it (D.N.); enrico.farnetti@ausl.re.it (E.F.); 16 Pathology Unit, Department of Medicine and Surgery, University of Parma, 43121 Parma, Italy

**Keywords:** PD-L1, prostate, cancer, *BRCA*, mismatch repair, microsatellite instability, *PTEN*, genes, immunotherapy

## Abstract

Pembrolizumab (anti-PD-1) is allowed in selected metastatic castration-resistant prostate cancer (PC) patients showing microsatellite instability/mismatch repair system deficiency (MSI-H/dMMR). *BRCA1/2* loss-of-function is linked to hereditary PCs and homologous recombination DNA-repair system deficiency: poly-ADP-ribose-polymerase inhibitors can be administered to *BRCA*-mutated PC patients. Recently, docetaxel-refractory metastatic castration-resistant PC patients with *BRCA1/2* or *ATM* somatic mutations had higher response rates to pembrolizumab. *PTEN* regulates cell cycle/proliferation/apoptosis through pathways including the AKT/mTOR, which upregulates PD-L1 expression in PC. Our systematic literature review (PRISMA guidelines) investigated the potential correlations between PD-L1 and MMR/MSI/*BRCA/PTEN* statuses in PC, discussing few other relevant genes. Excluding selection biases, 74/677 (11%) PCs showed dMMR/MSI; 8/67 (12%) of dMMR/MSI cases were PD-L1+. dMMR-PCs included ductal (3%) and acinar (14%) PCs (all cases tested for MSI were acinar-PCs). In total, 15/39 (39%) PCs harbored *BRCA1/2* aberrations: limited data are available for PD-L1 expression in these patients. 13/137 (10%) PTEN- PCs were PD-L1+; 10/29 (35%) PD-L1+ PCs showed PTEN negativity. *SPOP* mutations may increase PD-L1 levels, while the potential correlation between PD-L1 and ERG expression in PC should be clarified. Further research should verify how the efficacy of PD-1 inhibitors in metastatic castration-resistant PCs is related to dMMR/MSI, DNA-damage repair genes defects, or PD-L1 expression.

## 1. Introduction

Novel prognostic markers are urgently required to develop patient-tailored targeted-therapies in various tumors, including prostatic cancer (PC) [1,2,3,4,5,6,7]. In particular, increasing attention has been paid to immunohistochemical biomarkers such as Programmed death-1 (PD-1) and its ligand PD-L1, evaluating their ability to predict the response to immunotherapy drugs. PD-1 and PD-L1 are type-I transmembrane glycoproteins encoded by the *PDCD1* gene (located on chromosome 2) and *CD274* gene (located on chromosome 9), respectively: they are present on the surface of various immune cells, and PD-L1 may be expressed by tumor cells (including PC cells) [2,3,8,9,10,11,12,13,14,15,16,17,18,19,20,21,22,23,24,25,26,27,28,29,30,31,32,33,34,35,36,37,38,39,40,41,42,43,44,45,46,47,48,49,50,51,52,53,54,55,56,57,58,59,60,61,62,63,64,65,66,67,68,69,70,71,72,73,74,75,76,77,78,79,80,81,82,83,84,85,86,87,88,89,90,91,92,93,94,95,96,97,98,99,100,101,102,103,104,105,106,107,108,109,110,111,112,113,114,115,116,117,118,119,120,121,122,123,124,125,126,127,128,129,130,131,132,133,134,135,136,137,138,139,140,141,142,143,144,145,146,147,148,149,150,151,152,153,154,155,156,157]. PD-L1 expression on tumor cells is influenced by the tumor microenvironment, including the cytokines secreted by activated T cells and other immune cells (adaptive immune resistance) [4,6,8,9,10,11,12,13,15,33,36,70,71,76,100,101,108,130]. However, PD-L1 constitutive expression could also be induced by genetic alterations (such as PTEN loss) or activation of signaling pathways (intrinsic immune resistance); epigenetic regulators may also interfere with PD-L1 expression [4,6,8,9,10,11,12,13,24,58,71,125,126]. Limited data are available for PC [8,11,12,13,14,15,16,17,18,19,20,21,22,23,24,25,26,27,28,29,30,31,32,33,34,35,36,37,38,39,40,41,42,43,44,45,46,47,48,49,50,51,52,53,54,55,56,57,58,59,60,61,62,63,64,65,66,67,68,69,70,71,72,73,74,75,76,77,78,79,80,81,82,83,84,85,86,87,88,89,90,91,92,93,94,95,96,97,98,99,100,101,102,103,104,105,106,107,108,109,110,111,112,113,114,115,116,117,118,119,120,121,122,123,124,125,126,127,128,129,130,131,132,133,134,135,136,137,138,139,140,141,142,143,144,145,146,147,148,149,150,151,152,153,154,155,156,157].

The PD-1/PD-L1 pathway leads to the inactivation of PD-1 expressing cells, in particular CD8+ cytotoxic T cells, favoring tumor immune escape but also representing a target for immunotherapy drugs [2,3,8,9,10,11,12,13,14,15,16,17,18,19,20,21,22,23,24,25,26,27,28,29,30,31,32,33,34,35,36,37,38,39,40,41,42,43,44,45,46,47,48,49,50,51,52,53,54,55,56,57,58,59,60,61,62,63,64,65,66,67,68,69,70,71,72,73,74,75,76,77,78,79,80,81,82,83,84,85,86,87,88,89,90,91,92,93,94,95,96,97,98,99,100,101,102,103,104,105,106,107,108,109,110,111,112,113,114,115,116,117,118,119,120,121,122,123,124,125,126,127,128,129,130,131,132,133,134,135,136,137,138,139,140,141,142,143,144,145,146,147,148,149,150,151,152,153,154,155,156,157]. Despite some limitations of this approach, PD-L1 immunohistochemical expression is frequently tested in various tumor types to select patients for immunotherapy administration [158,159,160,161,162]. 

Pembrolizumab (anti-PD-1) is a highly selective IgG4-kappa humanized monoclonal antibody and immune checkpoint inhibitor; it binds with high affinity to the cell surface receptor PD-1 (expressed by T cells), blocking the inhibitory pathway, triggering a physiological shift to immune reactivity, and enhancing the antitumor immune response [4,9,23,26,34,64,156]. Recent evidence revealed good therapeutic activity of this drug in monotherapy, and the 2021 United States National Comprehensive Cancer Network (NCCN) guidelines have allowed pembrolizumab (as a second-line therapy or beyond) for selected patients with metastatic castration-resistant PCs (mCRPCs) showing high microsatellite instability/mismatch repair system protein deficiency (MSI-H/dMMR) or tumor mutation burden (TMB) >10 mutations/Mb [4]. In particular, the MSI/MMR status testing is recommended in mCRPC patients, and it may be considered in men with regional or castration-naïve metastatic PC [4]. For these reasons, in current practice, the International Society of Urologic Pathology recommends, if clinically indicated, MMR assessment via immunohistochemical analysis of MSH2, MSH6, MLH1, and PMS2 protein expression, with or without MSI testing, sequencing of MMR genes, and/or TMB estimate [6]. However, the dMMR/H-MSI status, as well as PD-L1 expression, may not necessarily correlate to the response to targeted immunotherapy, and combined analysis of MMR/MSI and PD-L1 status in PC patients was rarely performed [19,23,38,47,53,61,68,77,92,156]. 

TMB is a phenotypic hallmark of MSI-H/dMMR tumors [4]. Various genetic mutations, chromosomal aberrations, and molecular changes have been found in the genome of PC; however, most of them are still under investigation [10]. Germline mutations in MMR genes are drivers for the Lynch syndrome, which is an autosomal dominant genetic disorder causing multiple cancers (including PC) in affected patients [4]. In addition, the Breast Cancer 1-2 (*BRCA1/2*) genes are main regulators of a cellular DNA damage repair system (homologous recombination repair, HRR); the failure of HRR is due to combined germline and somatic mutations of *BRCA1/2* and/or other genes, favoring the activation of alternative and less effective DNA repair pathways (such as base/nucleotide excision repair or mismatch repair system) [7,8,9,19,23,53,56,92,163,164]. Indeed, *BRCA1/2* mutations are responsible for a group of hereditary PCs; specific drugs (poly-ADP ribose polymerase inhibitors, PARPi) can be administered to *BRCA*-mutated PC patients, according to the NCCN guidelines [4,9,19,23,53,56,92]. HRR gene testing is recommended for metastatic PC patients and considered for PC with spread to regional lymph nodes [4]. To improve the survival of mCRPC patients, treatment combinations (such as immunotherapy and PARPi) have been increasingly tested; however, few studies simultaneously tested PARPi and drugs affecting the PD-1/PD-L1 pathway; combined analysis of the *BRCA* and PD-L1 status was rarely conducted in PC patients [7,8,9,19,23,53,56,92].

We have performed a systematic literature review of the studies combining the analysis of PD-L1 expression with the investigation of the MSI/MMR, *PTEN*, and/or *BRCA* status in PC, trying to delineate the potential links between these markers in PC patients. The retrieved data concerning other main genes involved in PC genesis and progression are also discussed.

## 2. Materials and Methods

**Figure 1:**
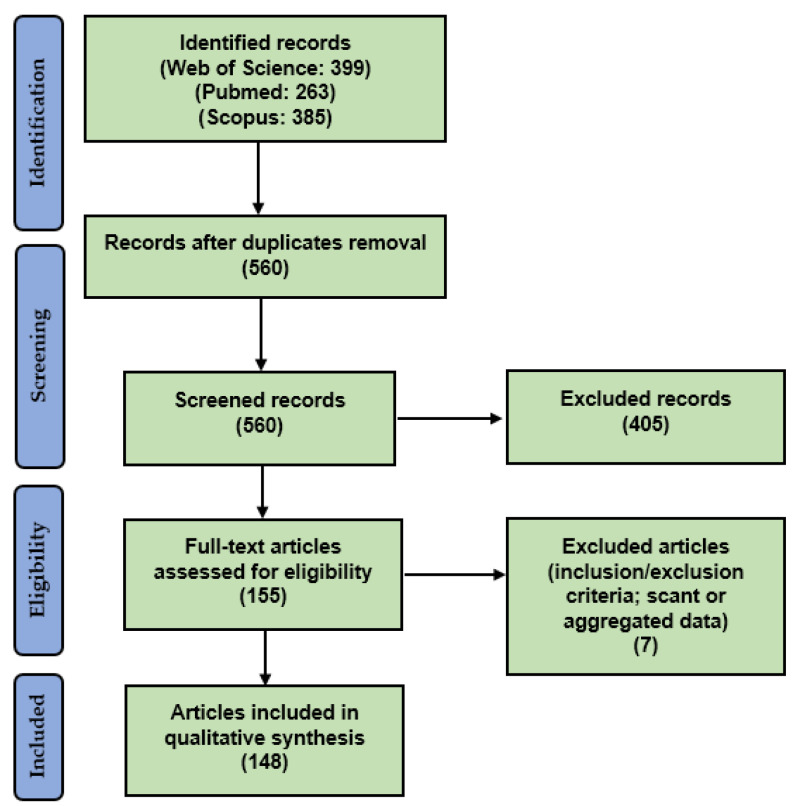
PRISMA flowchart of the systematic literature review.

Our systematic literature review was conducted according to the “Preferred Reporting Items for Systematic Reviews and Meta-Analyses” (PRISMA) guidelines (http://www.prisma-statement.org/, accessed on 8 May 2021) (Figure 1). 

We summarized the literature data concerning the role of PD-L1 in PC, describing the clinic–pathologic features of the published cases, and answering the following “Population, Intervention, Comparison, Outcomes” (PICO) questions:

- Population: patients, tumor cell lines, and mouse models included in studies concerning the role of PD-L1 in PC.
- Intervention: any treatment type.
- Comparison: no expected comparisons.
- Outcomes: patient’s status at last follow-up (no evidence of disease, alive with disease, dead of disease), response to therapy, overall survival (OS), progression-free survival (PFS), biochemical recurrence-free survival, metastasis-free survival (MFS), cancer-specific survival (CSS), disease-free survival, clinical failure-free survival.
- Our retrospective observational study satisfied the following:
- Eligibility/inclusion criteria: experimental studies (tumor cell lines, mouse models) or clinic–pathologic studies on human patients (case reports/series) concerning the role PD-L1 in PC.
- Exclusion criteria: non-prostatic tumors; non-carcinomatous histotypes; studies not examining PD-L1; uncertain diagnosis; review articles without new cases. No further exclusion criteria (including language or publication date) were applied.

We searched for (PD-L1 AND (prostate OR prostatic) AND (adenocarcinoma OR adenocarcinomas OR cancer)) in Web of Science (Topic/Title; 399 results; https://login.webofknowledge.com, accessed on 8 May 2021), Pubmed (all fields; 263 results; https://pubmed.ncbi.nlm.nih.gov, accessed on 8 May 2021), and Scopus (Title/Abstract/Keywords; 385 results; https://www.scopus.com/home.uri, accessed on 8 May 2021) databases. No limitations or additional filters were set. The bibliographic research ended on 8 May 2021. After duplicates exclusion, two independent reviewers screened the titles and abstracts of the 560 resulting records in order to verify the eligibility, inclusion, and exclusion criteria. The selected articles were obtained in full-text format: two other co-authors verified the relevance of the studies and searched for further pertinent references. Finally, two authors checked the extracted information. Globally, 155 full texts were considered for eligibility and, after reading them, seven articles were excluded as they were unfit according to the inclusion/exclusion criteria or because they presented scant or aggregated data; 148 articles were finally included [8,11,12,13,14,15,16,17,18,19,20,21,22,23,24,25,26,27,28,29,30,31,32,33,34,35,36,37,38,39,40,41,42,43,44,45,46,47,48,49,50,51,52,53,54,55,56,57,58,59,60,61,62,63,64,65,66,67,68,69,70,71,72,73,74,75,76,77,78,79,80,81,82,83,84,85,86,87,88,89,90,91,92,93,94,95,96,97,98,99,100,101,102,103,104,105,106,107,108,109,110,111,112,113,114,115,116,117,118,119,120,121,122,123,124,125,126,127,128,129,130,131,132,133,134,135,136,137,138,139,140,141,142,143,144,145,146,147,148,149,150,151,152,153,154,155,156,157]. 

Data collection was study-related (authors and year of study publication) and case-related (tumor stage at presentation, Grade Group, type of specimen, treatment, test methods, results of PD-L1 expression, follow-up and outcomes, experiment type).

The collected data were described as continuous variables (summarized by ranges, mean, and/or median values) or categorical variables (analyzed by frequencies and percentages).

As there was a lot of information to discuss, we have divided the presentation of our data into different articles, focusing on different sub-topics. Here, we analyze the studies of PC patients simultaneously evaluating PD-L1 expression and at least one of the following: status of mismatch repair system/microsatellite instability, *BRCA*, or *PTEN* genes. The potential correlations between PD-L1 expression and some of the other main genes involved in PC genesis and progression are also briefly discussed.

## 3. Results

### 3.1. Microsatellite Instability/Mismatch Repair System Deficiency and PD-L1 Expression in PC Patients

Table 1 reports the results of the studies on human PC patients, investigating both PD-L1 expression and the MMR/MSI status; supplementary data are reported in Table S1.

Moreover, the PD-L1 status of the cases included in the following studies was unclear:

- In the series of Abida et al. [48], 47/1551 (3%) PCs were classified as hypermutated (TMB ≥10 mutations/Mb). 32/1033 (3.1%) cases were MSI-H or dMMR (age: 39–85 years, median 64.5 years; 30 acinar, 1 ductal, and 1 small cell carcinoma; Gleason score 6–10; stage, N1 or M1; 21 mCRPCs, 67.7%); 7/32 (21.9%) carried pathogenic or likely pathogenic germline mutations in MMR genes (5 *MSH2*, 1 *MSH6*, 1 *PMS2*). One additional patient harbored a usually pathogenic germline *MSH6* mutation without MSI or hypermutation, while 7/1033 (0.7%) men with deleterious MMR gene alterations (1 germline, 6 somatic) did not have MSI or hypermutation. 11/32 (34%) MSI-H/dMMR PC patients received an anti–PD-(L)1 drug for mCRPC (as monotherapy or plus other immunomodulatory agents); 6/11 (54.5%) cases resulted in a >50% PSA decline, 4/8 (50%) evaluable cases achieved objective responses, 1 showed stable disease (6 months), and 3 progressed on radiographic exams.
- A phase I clinical trial [35] tested PT-112 (pyrophosphate conjugate) (alone or combined with avelumab, a PD-L1 inhibitor) on CRPCs. PSA declined in 6/14 (43%) cases (3 cases: ≥50% decrease); the PFS of a microsatellite stable responder was 11.3 months.
- McNeel et al. [65] found that 0/5 (0%) metastatic PC biopsies revealed a dMMR/MSI status; they were performed before vaccine treatment (alone or plus pembrolizumab).
- A CRPC patient (pM1; Gleason score 10, 5 + 5) was variably treated (leuprolide, abiraterone, prednisone; carboplatin + docetaxel; carboplatin + cabazitaxel + radiotherapy) before the administration of pembrolizumab + radiotherapy; after two cycles of pembrolizumab, an exceptional response was obtained, and the tumor revealed MSI-H and multiple pathogenic mutations in *AR, ATM, BRCA1, BRCA2, CDK12, PTEN*, and *TP53* (mean allelic frequencies: 0.23–7.2%) [26].

Globally, 280/883 (32%) PCs revealed MSI or loss of at least one MMR protein by immunohistochemistry (IHC) [9,19,23,29,37,38,43,53,68,77,80,92,156]. In particular, dMMR in ≥1 MMR protein was identified by IHC in 60/496 (12%) PCs [29,37,38,43,77,80]: MSH6 was the most frequently lost (45/60, 75%), followed by PMS2 (29/60, 48%), MSH2 (11/60, 18%), and MLH1 (3/60, 5%). At least two MMR proteins were lost in 21/496 (4%) cases [29,37,38,43,77,80] and ≥ 3 MMR proteins in 5/496 (1%) PCs [29,37], while all the four proteins resulted negative in 2/496 (0.4%) cases [29,37]. MSH6 IHC staining was indeterminate in two ductal PCs (MSH2 was also indeterminate in 1/2 cases) [43]. 

MSI was found in 210/263 (80%) PCs [9,19,23,53,68,92,156], including two cases with *MSH2* loss [19,53] and one with *MSH2/MSH6* deletion [19]. Four MSI cases revealed high TMB, while no further details were provided in the other cases [19,23,53,93]. 

It was usually unspecified how many dMMR/MSI PC patients harbored a Lynch syndrome. Some authors [61] found that germline mutations (*n* = 1), non-synonymous somatic mutations (*n* = 6), or biallelic events (*n* = 7) in MMR genes (total *n* = 14) had higher dMMR-associated DNA mutational signature activity and mRNA expression signatures. PCs with dMMR mutational signatures (*n* = 14) overexpressed a variety of immune transcripts, including CD200R1, BTLA, PD-L1, PD-L2, ADORA2A, PIK3CG, and TIGIT. 

According to our review, 29% acinar PCs, 7% ductal PCs, and 46% neuroendocrine carcinomas/tumors were PD-L1+ by IHC [8,11,12,13,14,15,16,17,18,19,20,21,22,23,24,25,26,27,28,29,30,31,32,33,34,35,36,37,38,39,40,41,42,43,44,45,46,47,48,49,50,51,52,53,54,55,56,57,58,59,60,61,62,63,64,65,66,67,68,69,70,71,72,73,74,75,76,77,78,79,80,81,82,83,84,85,86,87,88,89,90,91,92,93,94,95,96,97,98,99,100,101,102,103,104,105,106,107,108,109,110,111,112,113,114,115,116,117,118,119,120,121,122,123,124,125,126,127,128,129,130,131,132,133,134,135,136,137,138,139,140,141,142,143,144,145,146,147,148,149,150,151,152,153,154,155,156,157]. When data were analyzable, PD-L1 positivity was found in 214/273 (78%) MSI/dMMR cases [19,23,38,47,53,61,68,77,92,156].

As to the data presentation, it was difficult to understand the frequency and significance of MMR deficiency in PD-L1+ and PD-L1- groups. Sharma et al. [29,37] reported an association between the loss of ≥2 MMR proteins and higher PD-L1 expression rate in cancer cells (17.2% vs. 5.2%; *p* = 0.033; *n* = 127). Nava Rodrigues et al. [61] found a higher likelihood of PD-L1 positivity in MMR-deficient mCRPCs (5/10, 50%) compared to MMR-proficient cases (4/41, 9.8%) (*p* = 0.005); PD-L1 expression was associated with increased T-cells in mCRPCs (*p* = 0.007). Unfortunately, the study of Lin et al. [156] harbored a selection bias, as MSI-H and PD-L1 positivity in PC were inclusion criteria. Excluding this series (*n* = 206), only 74/677 (11%) PCs revealed MSI or loss of at least one MMR protein by IHC [9,19,23,29,37,38,43,47,53,61,68,77,80,92] and the PD-L1 positivity rate in MSI/dMMR cases was reduced to 12% (8/67 PCs); in particular, only 4/57 (7%) PCs revealed MSI [9,19,23,53,68,92] and just 1/35 (3%) MSI-PC was PD-L1+ [19,53,68], while only 2/9 (22%) dMMR PCs were PD-L1+ [38,43,77]. 

Subgrouping by histotypes, 57/402 (14%) acinar PCs and 3/94 (3%) ductal PCs were dMMR on IHC analysis (1 MSH2-/MSH6-; 1 MLH1-/PMS2-; 1 not otherwise specified) [38,43,80]. Moreover, MSH6 IHC staining was indeterminate in two ductal adenocarcinomas (MSH2 was also indeterminate in 1/2 cases) [43]. It was unclear if ductal PCs were both MMR-deficient and PD-L1+: 1/3 ductal PCs resulted PD-L1-, but the PD-L1 status of the other two cases was unspecified. All the cases tested for MSI seemed acinar PCs. Data of neuroendocrine carcinomas or carcinosarcomas were unavailable or unclear.

Somatic (or not specified as germline) aberrations in ≥1 DNA damage repair (DDR) genes were found in 3/5 (60%) MSI cases [19,23,53,56,93] and in 8/23 (35%) microsatellite stable (MSS) PCs [9,19,23,93] (Table S1). These aberrations involved *BRCA2* (2 MSI cases, 3 MSS cases) [9,19,56], *BRCA1* (1 MSI) [53], *ATM* (1 MSI, 2 MSS) [9,19,23,93], *TP53* (2 MSI, 2 MSS) [9,19,53], *CDK12* (1 MSS) [9], *FANCA* (1 MSS) [9], *FANCD2* (1 MSS) [9], *MLH3* (1 MSS) [9], *NBN* (1 MSS) [9], *RAD54L* (1 MSS) [9], and other genes. A MSI-PC showed *PTEN* mutation [53]. Some authors [53] found no association with the RNAseq rank of any tested genes, castration resistance vs. sensitivity status, or specimen type (primary vs. metastatic PC), nor between the DNA mutational profile, CD3/8 IHC status, RNA-seq CD8, PD-L1 IHC expression, or TMB and the expression profile of any genes (*n* = 17).

Few interesting results concerning the potential correlation of MMR/MSI with clinical outcome were reported in the studies resulting from our review. Some authors [61] found that dMMR was associated with decreased OS (*n* = 124; *p* = 0.005), while others (*n* = 220) [29,37] described that cases with loss of ≥1 MMR protein and PD-L1 expression in tumor-infiltrating lymphocytes (TILs) had a significantly higher risk of biochemical recurrence (BCR) (*p* = 0.045). No convincing association was identified as regards Gleason score, stage, age, or other variables. The reported series were usually retrospective, sometimes harboring selection biases and/or testing a few samples. Moreover, limited clinic–pathologic information was available in some studies, while the PD-L1 and MMR/MSI statuses were variably analyzed (different assays or antibody clones; variable scoring systems), and their potential correlation was rarely or unclearly investigated. Finally, studies were typically monocentric: larger validation cohorts from multiple hospitals are required.

As regards the response to therapy, PD-1 inhibition may be active in mCRPCs (including those tumors lacking MSI, DNA-repair defects, or PD-L1 expression), while not all the d-MMR/MSI-H cases respond to immunotherapy [9,19,23,48,92,156]. In the series of Abida et al. [48], 5/11 (45.5%) MSI-H/dMMR mCRPCs revealed durable clinical benefit upon continuous treatment, 5/11 (45.5%) cases had no benefit, while 1/11 (9.1%) PC revealed stable disease for 6 months; the PD-L1 status was unclear.

Graff et al. [23,92] found that only 1/5 (20%) responders to enzalutamide (ENZ) + pembrolizumab showed MSI, while 1/23 (4%) non-responders had a single MSI marker altered by polymerase chain reaction analysis (not deemed MSI-H by next-generation sequencing analysis, NGS), and 3/23 (13%) non-responders revealed heterozygous mutations in DDR pathways. As all the cases of Graff et al. were PD-L1-, there were no differences in PD-L1 expression (*n* = 10; *p* = 0.42) between responders and non-responders; molecular analysis found a higher number of neoepitopes among responders (679 vs. 405.5, *p* = 0.42) without differences in TMB [23,92]. 

None of the six responders to pembrolizuab showed MSI in another series [9]. Moreover, Petrylak et al. [19] analyzed the MSI status of 14 CRPCs: 1 dMMR (MSH2-/MSH6-) CRPC responded to atezolizumab, while another MSI-H PC did not benefit from treatment. However, the PD-L1 status of these cases was unclear, despite all the tested tumors showing absent expression or <5% positivity rate.

Lin et al. [156] recently reported a clinical trial including 206 men with previously untreated PD-L1+ (combined positive score, CPS ≥ 1) MSI-H-mCRPCs: pembrolizumab was administered alone (106/206, 51%; PA-group) or associated with ENZ (100/206, 49%; PE-group). In the first group, the PD-L1 CPS was 1–20 in 63%, 20–50 in 22%, and 50–100 in 15% of the cases, ranging from 1 to 20 in 64%, 20–50 in 22%, and 50–100 in 14% of PE-cases (*p* = 0.872). The median OS of PE-patients (vs. PA) was 28.6 vs. 21.3 months for CPS ≥ 50 (*p* = 0.001), 26.6 vs. 19.4 months for CPS ≥ 20 (*p* = 0.001) and 21.4 vs. 16.8 months for CPS ≥ 1 (*p* = 0.001): the benefit from PE therapy seemed more evident for higher PD-L1 expression in tumor cells. Regardless of the tumor mutation status, the addition of pembrolizumab to ENZ treatment was significantly associated with longer OS (median: 25.1 vs. 18.3 months; *p* = 0.001) and longer PFS (median: 6.1 vs. 4.9 months; *p* = 0.001) than pembrolizumab as monotherapy, despite the significantly major rate of adverse events (72% vs. 45%; *p* < 0.001).

### 3.2. PD-L1 and BRCA1/2 Status in PC Patients

Few studies analyzed PD-L1 expression and the status of *BRCA1/2* and other DDR genes in human PC patients (Table 2); additional information are reported in Table 1 and Table S1. 

Combining the scant analyzable data, aberrations in *BRCA1/2* genes were found in 15/39 (39%) cases [19,53,56], involving *BRCA1* (*n* = 1), *BRCA2* (*n* = 13), or both genes (*n* = 1). Moreover, 19/153 (12%) PCs of another series showed *BRCA1/2* or *ATM* mutations [9]. Unfortunately, the relation of PD-L1 expression with the *BRCA* status was unclear for most of the cases; only 1/10 (10%) PCs with *BRCA* aberrations was clearly PD-L1+ [19,53]. Some authors [53] found no association (*p* > 0.05) between the DNA mutational profile, CD3/8 or PD-L1 IHC status, RNA-seq CD8, or TMB, and the expression profile of any analyzed genes, including *BRCA1/2*. The scant available information did not allow us to discuss the potential differences in PD-L1 expression between PCs with somatic vs. germline *BRCA* mutations.

PD-1 inhibition has activity in mCRPCs, including tumors without MSI, DDR defects, or PD-L1 expression [23,92]. The phase II KEYNOTE-199 study reported that pembrolizumab showed antitumor activity and disease control with acceptable safety in docetaxel-refractory mCRPCs, regardless of the PD-L1 status, in both RECIST-measurable and non-measurable cases: 19/153 (12%) PCs had aberrations in *BRCA1/2* or *ATM*, while 10/153 (7%) PCs revealed alterations in ≥1 of 12 other HRR genes. Patients with somatic mutations in *BRCA1/2* or *ATM* showed higher response rates compared to the HRR-aberrant or HRR-normal groups (11%, 0%, and 3%, respectively). Unfortunately, it was not completely clear if there was a correlation between the *BRCA* and the PD-L1 status [165]. In another series [19], most of the CRPC patients with DDR genes alterations did not respond to atezolizumab (anti-PD-L1), and the DDR status was not a strong predictor of clinical benefit. Finally, some authors [56] included responders to durvalumab (anti-PD-L1) plus olaparib that harbored germline mutations in DDR genes, including *BRCA2* and *NBN*, but it was unclear if these cases were tested for PD-L1 by IHC.

### 3.3. PD-L1 and PTEN Status in PC Patients

Very few studies performed a combined analysis of PD-L1 expression and *PTEN* status in human PC patients by using molecular or IHC assays (Table 3) [19,38,53,77,86,96]; additional information is reported in Table 1, Table 2 and Table S1.

The assays and IHC scoring systems of PTEN and PD-L1 were variable and sometimes unclear. It was difficult to correlate the scant results in some cases; when data were analyzable, 179/326 (55%) PCs showed PTEN loss by IHC: 13/137 (10%) of PTEN- cases were PD-L1+, while 10/29 (35%) of PD-L1+ cases showed PTEN negativity [77,86,96]. In the series of Calagua et al. [77], the PD-L1^high^ and PD-L1^low^ components of the cases showing a nodular PD-L1 positivity pattern (*n* = 7; 39%) revealed a consistently concordant ERG status but a variable PTEN staining pattern. Conversely, Shaw et al. found no significant association between PD-L1+ tumors and ERG/PTEN status [38]. 

Only two studies evaluated PD-L1 IHC expression and performed molecular analysis of the *PTEN* gene, finding *PTEN* mutations (4/33, 12%) or copy number losses (5/33, 15%) (total: 9/33, 27%) [19,53]. The potential correlation between *PTEN* and PD-L1 status was usually unclear. Genomic analysis performed on a small series (*n* = 7) [53] reported no association (*p* > 0.05) between *PTEN* loss/mutation status with RNA-seq ranks of any gene, castration-resistance/-sensitivity, or primary vs. metastatic status, as well as no correlation (*p* > 0.05) of the DNA mutational profile, CD3/8 or PD-L1 IHC status, RNA-seq CD8, or TMB with the expression profile of any specific gene. 

In a Phase I trial [35] testing PT-112 (plus or without Avelumab) on CRPCs, a case with *PIK3CB* mutation and *PTEN* loss showed a 4-month response (93% PSA decrease, 48% reduction in target lesions); the PD-L1 status was unclear. 

### 3.4. PD-L1 and Other Genes Involved in PC Genesis and Progression

The *TP53* gene encodes 12 isoforms through alternative promoters, translation start sites, and RNA splicing [42]. In a study [42], increased expression of the Δ133TP53β isoform defined high-risk PC patients, while PCs showing increased Δ40TP53 and TP53α mRNAs levels revealed good prognosis. High Δ133TP53β mRNA levels alone predicted poor outcome (accuracy: 88%), correlating to advanced stage, increased proliferative index, immune cell infiltrate (T cells, macrophages, immunosuppressive cells), PD-1 overexpression on T cells, and PD-L1 upregulation in PC cells and CD163+ CSF1R+ macrophages [42]. Indeed, this isoform increased the expression of PD-L1 mRNA levels—while its knockdown reduced them—and of genes involved in immune signaling and migration. IL-6 was directly regulated by Δ133p53; its transcription may be induced by hypoxia in *T53*-wild-type tumors [42]. In a large series analyzing The Cancer Genome Atlas (TCGA) database, the immune class subtype of PCs revealed higher *TP53* mutation frequency than the non-immune class sub-group (14% vs. 10%) (especially vs. the immune-suppressed PCs: 19%, *p* < 0.001) [21]. *TP53* mutations were also found in 14 cases of our series [9,19,26,53], but the PD-L1 status of these cases was unclear.

Shaw et al. found no significant association between PD-L1 positivity and ERG status [38]. Conversely, Calagua et al. [77] reported a concordant ERG status among the PD-L1^high^ and PD-L1^low^ expressor PCs showing a nodular pattern of PD-L1 IHC positivity. ERG positivity was found in 16/44 (36%) PCs treated with neoadjuvant abiraterone acetate (ABT) + prednisone and leuprolide, and in 18/44 (41%) matched untreated PC controls (*p* = 0.5). ERG expression was also reported in 61/130 (47%) hormone-naïve PCs: five ERG+ PCs resulted PD-L1+ (*p* = 0.08). PD-L1+ PCs revealed a trend toward a lower rate of ERG positivity and higher androgen receptor (AR) expression. All the five PCs showing PD-L1 positivity in ≥25% of the tumor cells were ERG− [77]. Some authors reported *TMPRSS2-ERG* fusions in 7/17 (41%) PCs (3/7, 43%, also harboring *PTEN* aberrations), as well as *SPOP* (*n* = 1), *TP53* (*n* = 6), or *RB* (*n* = 1) mutations, or *RB* copy number loss (*n* = 3) (Table 1, Table 2 and Table 3) [53]. However, it was not completely clear which cases resulted in PD-L1 positivity. A partial responder to atezolizumab harbored *TMPRSS2-ERG* fusion, *MSH2/MHS6* deletion, high TMB (30 mutations/Mb), as well as *BRCA2* E49*, *TP53* Y236D, *AR* W742C mutations, and *MYC* amplification; in this case, PD-L1 positivity was found in ≥5% immune cells and <5% of tumor cells after treatment, while the previous baseline biopsy was negative [19]. 

In human tumors, mutations in the PD-L1 C-tail are mutually exclusive with those in the substrate-interacting N-terminal meprin and TRAF homology (MATH) domain of Speckle-type POZ protein (SPOP) [75]. SPOP promotes ubiquitin-mediated PD-L1 degradation; in a series (*n* = 97; 15 *SPOP^mutant^* and 82 *SPOP^wild-type^*), SPOP-deficient PCs correlated with increased PD-L1 expression and a decreased number of CD8+ TILs [75]. *SPOP^mutant^* cases more frequently showed strong PD-L1 IHC positivity (80% vs. 10%), while 70% of *SPOP^wild-type^ PCs* revealed weak/absent PD-L1 staining [75]. In a large series, the immune-suppressed PC group showed fewer *SPOP* mutations than the immune-activated and non-immune class (5.6%, 13.5%, and 13.2%, respectively, *p* < 0.001) [21]. Other authors [19] reported *SPOP* and *MYC* mutations in one and four cases, respectively: the PD-L1 status was unclear. Further studies are required. 

## 4. Discussion

### 4.1. PD-L1 and dMMR/MSI

The MMR system is a post-replicative, high-fidelity, single-strand repair mechanism, identifying and reversing the DNA base mismatches and insertion/deletion (indel) loops; MMR deficiency occurs through mutational or epigenetic events, resulting in MSI and hypermutator phenotypes associated with chemoresistance and immunotherapy sensitivity [9,19,23,29,37,38,43,47,53,61,68,77,80,92,166]. Hypermutation is correlated to the higher expression of tumor neoantigens, favoring immune recognition [167,168]. 

According to the literature, about 1–12% of mCRPC are MSI-H/dMMR [4,37,38,39]. In our review, about 11% PCs globally revealed MSI or the IHC loss of at least one MMR protein [9,19,23,29,37,38,43,53,68,77,80,92], while PD-L1 positivity was identified in about 12% of dMMR/MSI PCs [19,23,38,47,53,61,68,77,92] after excluding a study with relevant selection bias [156]. Only 4/57 (7%) PCs clearly revealed MSI [9,19,23,53,68,92], and just 1/35 (3%) MSI-PC was PD-L1+ [19,53,68]. Subgrouping by histotype, 57/402 (14%) acinar PCs and 3/94 (3%) ductal PCs (high-grade PCs) of our review were dMMR, while all the cases tested for MSI apparently seemed acinar PCs [8,11,12,13,14,15,16,17,18,19,20,21,22,23,24,25,26,27,28,29,30,31,32,33,34,35,36,37,38,39,40,41,42,43,44,45,46,47,48,49,50,51,52,53,54,55,56,57,58,59,60,61,62,63,64,65,66,67,68,69,70,71,72,73,74,75,76,77,78,79,80,81,82,83,84,85,86,87,88,89,90,91,92,93,94,95,96,97,98,99,100,101,102,103,104,105,106,107,108,109,110,111,112,113,114,115,116,117,118,119,120,121,122,123,124,125,126,127,128,129,130,131,132,133,134,135,136,137,138,139,140,141,142,143,144,145,146,147,148,149,150,151,152,153,154,155,156,157]. Data of other aggressive histotypes (such as neuroendocrine carcinomas or carcinosarcomas) were unavailable or unclear; further studies are required.

MMR deficiency could be associated with aggressive clinic–pathologic features in PC (high Gleason score, advanced stage, decreased OS); dMMR mutational signatures were sometimes associated with increased T cell-related and checkpoint-related transcripts (including PD-L1 and PD-L2) [61,167,168]. In another series [29,37], loss of ≥1 MMR protein and PD-L1 expression in TILs had a significantly higher BCR risk. 

An association between MSI-H status and the efficacy of PD-L1/PD-1 inhibitors has been established in a range of solid tumors, including a relatively small number of PC cases; MMR deficiency was sometimes associated with favorable response to anti-PD-1 therapy or PD-L1 expression [29,37,61,167,168,169,170,171,172,173,174]. The 2021 NCCN guidelines have allowed immunotherapy in men with asymptomatic or minimally symptomatic mCRPCs; pembrolizumab can be administered as ≥2nd line systemic therapy to dMMR/MSI-H mCRPCs that have progressed through prior docetaxel and/or novel hormone therapy [4]. Durable clinical benefit, sometimes with a 50% reduction in PSA levels, was occasionally reported in PC patients receiving checkpoint inhibitors as monotherapy or combination therapy, although with variable response rates: it was usually unclear if PD-L1 was tested in all these cases [48,167,175]. However, the dMMR/H-MSI status does not necessarily correlate to the response to immunotherapy: emerging clinical data have reported that dMMR/MSI-H mCRPCs do not always respond, while responders are not necessarily dMMR/MSI-H (by IHC and/or polymerase chain reaction analysis) [48,61].

The NCCN guidelines recommend using a metastatic biopsy for histologic and molecular analysis of somatic tumor testing [4]. Indeed, the MSI-H/dMMR phenotype may be acquired somatically during disease evolution; in a study, MSI may have been subclonal in earlier samples of two longitudinally profiled PCs, which is probably due to tumor heterogeneity (not favoring a truncal event) [48]. Unfortunately, the NCCN guidelines [4] do not define how to evaluate the MSI-H/dMMR or the PD-L1 status: MSI-H or dMMR can be tested by using either DNA tests or IHC. The second approach is easy to use and cost-effective, but it may be insufficient to predict PC recurrence after radical prostatectomy [29,38,61,168,169,170,171,172,173,174]. In case of molecular testing, validated NGS assays are preferred by the NCCN guidelines [4]. NGS can assess for the MSI-H/dMMR status by interrogating microsatellite loci to identify MSI, finding mutations and copy number alterations in MMR-associated genes; it can also infer the TMB, which is a phenotypic hallmark of MSI-H/dMMR tumors [176,177]. In clinical practice, data on TMB and MMR genes mutational status could help in case of indeterminate MSI sensor scores or low-quality/purity tumor tissues [171,172]. 

About 40–50% of PCs are hereditary, frequently showing earlier onset, familial clustering, and multifocality; mutations and polymorphisms of several tumor suppressor genes and proto-oncogenes play a key role in PC onset and progression [10,169]. The impairment of MMR genes has been linked not only to sporadic PC cases (usually harboring *MSH2* or *MSH6* gene defects), but also to some hereditary forms of PC [4,10,169,178,179]. Lynch syndrome is an autosomal-dominant genetic disorder driven by germline mutations in MMR genes (such as *MLH1*, *MSH2*, *MSH6*, and *PMS2*), harboring greater cancer risk (especially for colorectal adenocarcinoma, but also for PC) through MSI [4,10,169,178,179]. NGS may identify germline MMR mutations, and genetic counselling for Lynch syndrome is advised for MSI-H/dMMR patients by the NCCN guidelines [4]. Unfortunately, the scant available information did not allow us to understand how many cases really harbored a Lynch syndrome and to discuss the potential differences in PD-L1 expression between somatic vs. germline MMR genes mutations.

NGS may also represent an efficient strategy to identify PC patients who may benefit from anti-PD-1/PD-L1 therapies, especially in cases with higher TMB (often associated with MMR genes alterations) [4,30,61,176]. In various neoplasms, higher TMB seems to predict favorable outcomes related to anti-PD-1/PD-L1 immunotherapy administration; indeed, the 2021 NCCN guidelines have also allowed pembrolizumab (as a second-line therapy or beyond) for mCRPCs, showing TMB > 10 mutations/Mb [4]. In PC, the median PC TMB is 3.6–4 mutations/Mb, while only 2–7.7% of cases have >20 mutations/Mb [30,61,177,180]. CRPCs usually show low TMB with alterations in key regulatory pathways (such as *PI3K* and androgen pathways), while ≈50% of CRPCs carry gene fusions correlating with poor prognosis: fusion-positive PCs may still harbor many neoantigens from gene fusions, potentially serving as immunotherapy targets [30,181,182]. In a PC series [30], high tumor fusion burden (TFB)—measuring the number of gene fusions in a tumor—inversely correlated with TMB and immune suppressive signatures; conversely, it was positively associated with immune infiltration, PD-L1 expression on immune cells, and immune signatures (representing activation of T cells and M1 macrophages, checkpoint inhibitors, IFN-γ-induced T-cell activity, T-effector activation, and class I antigen processing), cell cycle progression, AR signaling, and ERG/ETS transcriptional activity. Cases with *ERG* fusion had significantly higher overall fusion burden (*p* < 0.0002) and higher *ERG* transcriptional activity (*p* < 0.0001) [30]. On the other hand, Chen et al. reported that PD-L1, CD8A, or CYT expression was not associated with TMB and neoantigen number in PC [76].

### 4.2. PD-L1 and BRCA1/2

*BRCA1* regulates the cellular DNA damage response and repair, transcriptional regulation, and chromatin modeling, while *BRCA2* plays a role in the DDR processes [9,19,23,53,56,92,163]. In PC, *BRCA1* co-regulates the AR activity mediating tumor progression, while *BRCA2* limits the metastatic potential of PC by MMP9 downregulation and inhibition of the *PI3K/AKT* and *MAP/ERK* pathways (which also regulate PD-L1 expression) [163,164,183,184]. 

**Figure 2:**
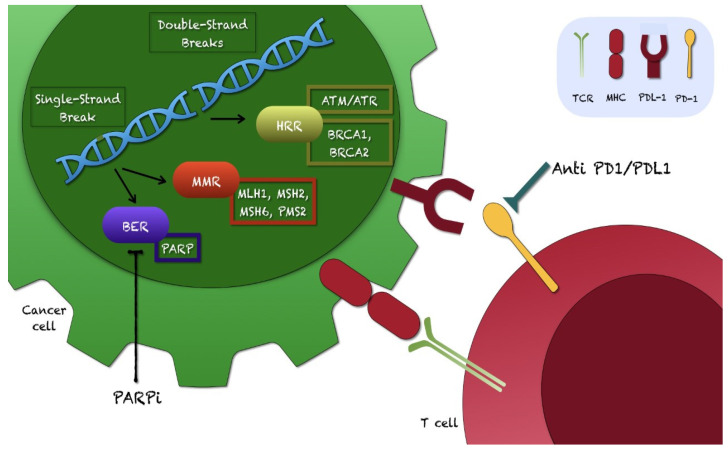
Intracellular DNA damage repair pathways (BER: base excision repair; HRR: homologous recombination repair; MHC: major histocompatibility complex; MMR: mismatch repair system protein; PARP: poly-ADP-ribose polymerase; PARPi: PARP inhibitors; TCR: T-cell receptor).

Loss of *BRCA1* or *BRCA2* is linked to a deficiency in HRR of DNA double-strand breaks; the failure of the *BRCA*-mediated HRR mechanism is due to combined germline and somatic mutations, favoring the activation of alternative and less effective DNA repair pathways (such as base/nucleotide excision repair or mismatch repair system) (Figure 2) [7,8,9,19,23,53,56,92,163,164].

Initial studies estimated a <15% *BRCA1-2* mutation rate in PCs [169,185,186,187,188,189,190,191]. Some authors found that 1–2% of young-onset PC patients had a germline *BRCA2* mutation, conferring a five to seven-fold greater lifetime risk for developing PC (three to eight-fold greater risk for *BRCA1* mutations) [10,192]; other studies estimated a 20% lifetime risk for *BRCA2*-mutated patients (9.5% for *BRCA1*-mutated carriers) [169,186]. The TCGA (*n* = 333 PCs) identified several germline mutations of DDR genes, including *BRCA2* (13%), *ATM* (7.3%), *MSH2* (2%), and *BRCA1* (0.3%) [189]. More recently, other authors found higher *BRCA2* (24.3%) and *BRCA1* (6.4%) germline mutation rates (6.4%) [190]; mCRPCs may present up to 20–30% of DDR genes mutations [191].

Loss of function of *BRCA1/2*, *ATM* (a DNA-damage checkpoint indirectly activating HRR), and other HRR genes are associated with more aggressive PCs (higher Gleason score; lymph node involvement, and/or metastatic disease at presentation; worse CSS/MFS) [169,170]. In a study, the CSS of *BRCA*-mutated PC patients was 8.6 years compared to 15.7 years in non-carriers (MFS 77% vs. 93%) [187]. Compared to non-carriers, *BRCA2* mutation carriers showed a higher rate of >50% PSA decline (Pomerantz et al.: 75% vs. 17%; Schmid et al.: 64% vs. 20–30%) and OS after platinum-based therapy (Pomerantz et al.: 18.9 vs. 9.5 months) [193,194].

Very few studies simultaneously analyzed PD-L1 expression and *BRCA* status [9,19,23,53,56,92]. *BRCA1/2* genes aberrations were found in 15/39 (39%) PCs [19,53,56], but the PD-L1 status was usually unclear, as only 1/10 (10%) PC with *BRCA* aberrations was clearly PD-L1+ [19,53]. The scant available information did not allow the discussion of the potential differences in PD-L1 expression between somatic vs. germline *BRCA* mutations.

*BRCA1/2* mutations also affect the response to treatment; metastatic PC patients with germline *BRCA2* mutations became resistant to the androgen deprivation therapy faster than non-carriers (13.2 vs. 28 months), showing a halved CSS [9,19,23,53,56,92,188]. Occasional studies investigated if the BRCA/DDR genes status may also affect the response to immunotherapy [19,23,92,170]. Petrylak et al. reported that most CRPC patients with DDR alterations did not respond to atezolizumab (anti-PD-L1): the DDR status was not a strong predictor of clinical benefit [19]. However, in other studies, PD-1 inhibition revealed activity in mCRPCs, including tumors lacking MSI, DDR defects, or PD-L1 expression [23,92]. In the phase II KEYNOTE-199 study, pembrolizumab (anti-PD-1) showed antitumor activity and disease control with acceptable safety in docetaxel-refractory mCRPCs, regardless of PD-L1 status, in both RECIST-measurable and non-measurable cases; 19/153 (12%) tested PCs had *BRCA1/2* or *ATM* aberrations and higher responses rates, but it was unclear if there was a correlation between the *BRCA* and PD-L1 status [170]. Further studies are required.

PARPi are Food and Drug Administration (FDA)-approved drugs for *BRCA*-mutant ovarian and breast carcinomas [9,19,23,53,56,92,195]. PARPi block the base excision DNA repair pathway (Figure 2) while defects in *BRCA* simultaneously prevent HRR, leading to genomic instability and the accumulation of DNA damage; these effects can trigger cell cycle arrest and apoptosis [195]. Olaparib and rucaparib are PARPi approved for the treatment of mCRPCs with *BRCA* germline mutations, while new drugs are under investigation (niraparib and talazoparib) [191,196]. In a clinical study, compared to ABT alone, olaparib plus ABT improved the PFS (8.2 vs. 13.8 months) [197], which was probably regardless of the mutational status of HRR genes [129]. Rucaparib induced PSA and radiographic responses in 48% and 45%, respectively, in *BRCA2* mutation carriers (phase II trial) [196], while olaparib showed better PFS than AR signaling inhibitors (ARSi) in mCRPC patients with *BRCA1-2* germline mutations (7.4 vs. 3.55 months) (PROFOUND phase III trial) [191]. In mCRPCs having HRR genes alterations and progressing on ENZ or ABT, olaparib was associated with longer PFS and better response to therapy than ENZ/ABT alone, while niraparib revealed a higher objective response rate (ORR) (41% vs. 9%) and longer PFS (8.2 vs. 5.3 months) in mCRPC patients with DDR defects receiving ARSi and taxane-based chemotherapy [197,198,199]. The TALAPRO-1 phase II trial (NCT03148795; https://clinicaltrials.gov, accessed on 26 December 2021) is evaluating talazoparib in mCRPC patients: initial data reported better ORR for *BRCA1-2* (43.9%) vs. *ATM* (11.8%) mutations carriers [191,200]. 

Exceptionally, some authors performed a combined analysis of the effects of anti-PD-1/PD-L1 and PARPi in PC patients [56]. Durvalumab is a human anti-PD-L1 IgG1-K monoclonal antibody approved by the FDA for locally advanced or metastatic urothelial cancer and locally advanced, unresectable, stage 3 non-small cell lung cancer; Karzai et al. [56] found that olaparib (PARPi) plus durvalumab demonstrated activity in PC patients without biallelic inactivation of DDR pathways, reaching deep responses in mutated cases: this drug combination revealed acceptable toxicity and efficacy. It was unclear which cases were evaluated for PD-L1. Further studies are required.

Genotoxic stress and stalled DNA replication forks favor the expression of ligands for the NKG2D receptor of NK cells [195]; DDR inhibitors may also increase the NK killing activity [129]. In PC cell lines, olaparib significantly increased tumor cell sensitivity to NK-mediated killing and antibody-dependent cytotoxicity (ADCC), regardless of *BRCA* status, PD-L1, or epithelial growth factor receptor (EGFR) modulation [129]. PARPi activates the “stimulator of interferon genes” (STING) pathway in breast carcinoma, upregulating PD-L1; in *BRCA* wild-type PC cell lines, STING upregulation occurred after PARPi administration without increasing the PD-L1 expression [129]. Conversely, STING was not expressed in *BRCA* mutant 22RV1 DU145 PC cell lines, either before or after olaparib treatment; disparities may be due to differences in olaparib exposure [129]. 

Olaparib may enhance the killing activity of endogenous or engineered high-affinity NK cells [107,129]. ADCC is elicited by the interaction between CD16 (FcγRIII) on NK cells and the Fc portion of IgG1 antibodies of target cells; the NK-mediated immune surveillance and killing of non-self cells are induced by target cell surface death receptors, activating a caspase cascade and resulting in apoptosis [107,129]. In PC cell lines, olaparib upregulated TRAIL-R2 (a death receptor targeted by the TRAIL ligand on NK cells), activating the caspase cascade [129]. The combined use of NK- and ADCC-mediating agents with PARPi in *BRCA* mutant and wild-type PC may improve treatment efficacy. A lack of PD-L1 upregulation suggests that ADCC-mediating antibodies not targeting PD-L1 (such as cetuximab) may also be exploited in combination with PARPi [107,129].

### 4.3. PD-L1 and PTEN

PD-L1 expression on tumor cells can be induced by cytokines secreted by activated T cells (favoring the adaptive immune resistance) or due to constitutive expression induced by genetic alterations (such as *EGFR/KRAS* mutations, *ALK* rearrangements, *PTEN* loss) or activation of signaling pathways (intrinsic immune resistance) [10,19,38,53,77,86,96,200].

*PTEN* is a tumor suppressor gene encoding the phosphatase and tensin homolog, a lipid and protein enzyme. It regulates cell cycle, proliferation, and apoptosis through different pathways, including the PI3K/AKT/mTOR (which upregulates PD-L1 in PC) [10]. 

PTEN loss and the associated *PI3K* gene activation correlated to PD-L1 expression in tumors such as glioblastoma [201,202,203]. *PTEN* activity may be also reduced in PC; its expression can lack in whole tumor or in some areas [10,19,38,53,77,86,96]. *PTEN* loss is a relatively frequent, late event in mCRPCs, which is probably associated with worse prognosis [197,204,205]; however, it may indicate a sensitivity to Akt-inhibitors (such as ipatasertib) [200,206]. 

According to our review, 179/326 (55%) PCs showed PTEN loss by IHC: 13/137 (10%) of PTEN- cases were PD-L1+, while 10/29 (35%) of PD-L1+ cases were PTEN- [77,86,96]. The few studies investigating PD-L1 and PTEN IHC expression did not find significant correlations [38,77]; however, various (sometimes unclear) assays and scoring systems of PTEN and PD-L1 were used, and few cases were tested. PTEN loss may increase the levels of several immunosuppressive cytokines, as well as the infiltration of granulocytic myeloid-derived suppressor cells, and the inhibition of T-cell–mediated tumor killing; it can also decrease the T-cell trafficking into the tumor microenvironment [107]. 

*PTEN* expression can be altered by gene deletion (bi- or mono-allelic), DNA methylation, transcriptional repression, and translational disorder, possibly leading to distinctive signaling modulations [107]. *PTEN* mutations (4/33, 12%) or copy number loss (5/33, 15%) were found in two molecular studies (total: 9/33, 27%) [19,53], but correlations between the PTEN and PD-L1 statuses were not reported in detail. 

The PI3K/AKT/mTOR pathway contributes to regulate PD-L1 expression in PCs [8,11,12,13,14,15,16,17,18,19,20,21,22,23,24,25,26,27,28,29,30,31,32,33,34,35,36,37,38,39,40,41,42,43,44,45,46,47,48,49,50,51,52,53,54,55,56,57,58,59,60,61,62,63,64,65,66,67,68,69,70,71,72,73,74,75,76,77,78,79,80,81,82,83,84,85,86,87,88,89,90,91,92,93,94,95,96,97,98,99,100,101,102,103,104,105,106,107,108,109,110,111,112,113,114,115,116,117,118,119,120,121,122,123,124,125,126,127,128,129,130,131,132,133,134,135,136,137,138,139,140,141,142,143,144,145,146,147,148,149,150,151,152,153,154,155,156,157]. Activating *PIK3CA* mutations increased PD-L1 expression in PC, while *RAS/MAPK* activating mutations may represent a “second hit” to the PTEN/PI3K/AKT pathway loss in mCRPCs [202,203]. To our review, *PTEN* upregulation with consequent inhibition of mTOR and PD-L1 expression has been documented in mice injected with PC cells treated with recombinant human chemerin (chemoattractant protein and *PTEN* activator) [107]. The chemerin-induced AKT-mTOR and PD-L1 downregulation significantly reduced PC growth: *CMKLR1* knockdown abrogated this pathway. These data revealed a potential *CMKLR1/PTEN*/PD-L1 signaling cascade that may occur through the PI3K/AKT/mTOR pathway (as also suggested by experiments with targeted inhibitors) [105,107]. In a study [107], the PC cells of a patient also expressed CMKLR1: as in PC cell lines, chemerin may act through CMKLR1 on tumor cells to modulate PTEN and PD-L1. The type of *PTEN* loss may dictate the relevance of chemerin modulation in humans [107]. In PC cell lines with complete allelic *PTEN* loss, modulating tumor chemerin levels did not result in changes in PD-L1 expression by the tumor; however, chemerin may recruit immune effector cells into the tumor microenvironment, still having benefits in outcomes [107]. Conversely, in PCs with intact (but decreased) *PTEN* expression, chemerin modulation may decrease PD-L1, suppressing tumor growth and improving responses to immunotherapy [107]. 

Analyzing PCs of *PTEN*-null mice, some authors reported that IL-17rc wild-type mice showed higher levels of PD-1, PD-L1, and PD-L2, developing more invasive PCs than IL-17rc knockout mice [150,151,152]. Combined treatment with anti-PD-L1 antibody (clone D265A, mouse/IgG1 kappa) and AZD1480 (JAK1/2 inhibitor), followed by androgen deprivation therapy, improved the antitumor immune responses over monotherapy in *PTEN*-knockout mice, potentially decreasing the immunosuppressive effects of androgen withdrawal [142]. Shimizu et al. reported that *PTEN/P53-DKO* mice with advanced PCs tended to longer OS after treatment with anti-PD-L1 drugs [138].

Unfortunately, the relationship of the innate immune resistance (whereby PD-L1 is constitutively upregulated when *PTEN* is lost) has not been explored in depth in PC patients. In a few series, PD-L1 expression was independent from *PTEN* status [38,77,96] or *PI3K* pathway activation [96]. Further larger studies on human patients have to investigate the potential correlation between *PTEN* and PD-L1.

### 4.4. Comments on Some Other Genes Involved in PC Carcinogenesis and Progression

*TP53* is a tumor-suppressor gene encoding a nuclear transcription factor (p53). In case of DNA damages, p53 blocks cell cycle progression, regulating the G1/S and G2/M checkpoints [42,207]. *TP53* gene mutations can adversely affect PC prognosis; they seem more common in advanced stage, metastatic, and/or androgen-independent PCs [42,207]. The *TP53* gene encodes 12 isoforms through alternative promoters, translation start sites, and RNA splicing. The Δ133p53 isoform has pro-tumorigenic functions, favoring cell cycle progression, anti-apoptotic activity, angiogenesis, migration, increased DNA repair and telomerase activity, and reduced chemosensitivity; it may also promote inflammation in cancer [42]. In a study [42], increased expression of the Δ133TP53β isoform seemed to define high-risk PC patients. Other authors [208] reported that only the number of active mast cells showed significant differences between low- and high-risk prognostic groups of *TP53*-mutated PCs [208]. 

The tumor-suppressor *RB* gene encodes a nuclear transcription factor regulating the cell cycle at the G0/G1 phase, DNA damage response, checkpoint activation, and differentiation [10,55]. At the end of the mitotic phase, the RB protein is dephosphorylated; in this active form, it interacts with E2F transcription factors and inhibits the G1/S phase transition [55]. Upon mitogen stimulation or in the late G1 phase, cyclin-dependent kinases (CDKs) phosphorylate and inhibit RB, resulting in E2F factors release from RB sequestration and allowing progression to the S phase [55]. RB may also have E2F-independent tumor-suppressor functions [209]. *RB* mutations allow cell cycle progression; it is controversial if the RB/p16 pathway loss is an early or late event in PC [10]. Experimental studies suggested that *RB* deletion could be associated with PD-L1 overexpression in mCRPCs [55]. RB binds to the NFκB protein: in PC cell lines, CDK4/6-phosphorylated RB may promote cancer immunity through inhibition of NF-kB transcriptional activity and of PD-L1 expression [55]. CDK4/6-inhibitors, *RB* deletion, and irradiation can induce PD-L1 upregulation, thus causing immune evasion of PC cells [55]. A small bioactive RB-derived serine-249/threonine-252 phosphorylation-mimetic peptide decreased PD-L1 expression via NF-kB inhibition and by enhancing the anti-cancer efficacy of radiotherapy; it blocked the p65 binding to the cognate DNA sequence in the PD-L1 promoter [55]. Upon stimulation of proinflammatory cytokines, the MAP3K7–IKK signaling axis activates the transcription factor NF-kB, which regulates pro-survival genes and PD-L1 mRNA expression in various cancer types (including PC), probably favoring cancer immune escape [55].

*c-MYC* regulates cell growth and proliferation, cycle progression, transcription, differentiation, and apoptosis. *c-MYC* amplification leads to high-grade, metastatic, and/or androgen-resistant PCs [10]. However, *c-MYC* activation may also appear in early phases of PC [210]. In PC experimental studies, *c-MYC* expression and/or the combined deletion of *PTEN/SMAD4* or *PTEN/TP53* expanded immunosuppressive tumor-associated macrophages and myeloid-derived suppressor cells, promoting tumor immunotolerance and vascularization [132]. Moreover, *c-MYC* seems involved in PD-L1 regulation. The MYC inhibitor 361 (MYCi361) suppressed the tumor growth of MycCaP PC cell lines in mice, increased the tumor immune cell infiltration, upregulated PD-L1 on tumor cells, and sensitized cancer to anti-PD-1 immunotherapy [211]. In PC cell lines, the IFN-γ-induced PD-L1 mRNA and protein levels were significantly abrogated by knockdown of the histone methylation regulators WDR5 or MLL1 (not by *C-MYC* silencing); WDR5 seemed important for PD-L1 transcription, while OICR-9429 (antagonist of WDR5 interactions with MLL1, C-MYC, and other partners) blocked this process [10]. OICR-9429 and WDR5 knockdown also significantly reduced c-Myc recruitment [10,12]. 

The *ERG* gene encodes for a transcription factor of the ETS family; it is fused with the prostate-specific and androgen-responsive *TMPRSS2* (transmembrane protease, serine 2) gene in about 50% of PCs, resulting in ERG overexpression. Two other *ERG* gene fusions (*SLC45A3*:*ERG*, *NDRG1*:*ERG*) can increase ERG expression, despite them occuring in <5% of PCs [166]. The *ETS-TMPRSS2* fusions are apparently mutually exclusive from certain genomic aberrations: *RAF-RAS-FGFR* gene fusions may occur only in ETS-negative tumors [166,212]. ERG overexpression is a driver event in the transition from prostatic intraepithelial neoplasia to carcinoma [212]. Moreover, the high expression of ERG seemed associated with PCs showing advanced stage, high Gleason score, metastatic behavior, and/or shorter survival [213]. Androgen-driven *ERG-TMPRSS2* gene fusions were associated with disease recurrence and tumor relapse [212,213]. Although *ERG* fusions/overexpression may have a diagnostic and predictive role for aggressiveness in PCs, few studies investigated their relationship with the PD-L1 status. Some authors found no significant association between PD-L1+ tumors and ERG status [38], while others reported a concordant ERG status with a nodular pattern of PD-L1 IHC positivity [77]. A trend of PD-L1+ PCs toward a lower rate of ERG positivity and higher AR expression was suggested [77]. Further data are required. The bromodomain and extraterminal (BET) family of proteins are transcriptional coactivators of cell cycle, apoptosis, migration, and invasion, frequently enhancing the expression/transcription of oncogenic drivers, such as *AR* and *ERG* in PC [213]. In PC cells lines, ERG negatively regulates miR-200c expression [86]. In a large series, miR-200a-c positively correlated to PD-L1 mRNA levels, being inversely associated with methylation of the PD-L1 promoter [86]. 

*SPOP* mutations occur in about 10–15% PCs, representing potential predictors of CRPC response to ABT [163]. These mutations are largely clustered within the MATH domain, which is responsible for substrate recognition and interaction, while the C-terminal BTB domain binds CUL3, forming the functional E3 ubiquitin ligase complex. *SPOP* mutations have a dominant-negative effect on substrate binding and degradation, being mutually exclusive with *ERG* gene fusions (the most frequent genetic alterations in PC); they increase the mRNA and protein levels of ERG and its downstream targets, promoting cellular migration and invasion [2]. Both the MATH and BTB domains are required for the SPOP-mediated BRD4 ubiquitination and degradation [2,214]. In PCs, *SPOP* mutations confer resistance to BET inhibitors through the stabilization of BRD4. Mutations at the E3 ligase (SPOP) or the substrate (BRD4) may inhibit the SPOP-mediated BRD4 destruction by disrupting the SPOP–BRD4 interaction, stabilizing BRD4, and leading to its cooperation with AR, ERG, and other oncogenic transcription factors [214]. Cyclin D-CDK4 kinase destabilizes PD-L1 via culliculin 3-SPOP to control tumor surveillance: proteasome or ubiquitin E3 ligase inhibitors incremented PD-L1 expression [75]. Cancer-derived *SPOP* mutants failed to promote PD-L1 degradation by poly-ubiquitination because of their deficiency in binding to PD-L1 [75]. In human tumors, *SPOP* mutations in the PD-L1 C-tail are mutually exclusive with those in the substrate-interacting MATH domain [75]. SPOP can promote ubiquitin-mediated PD-L1 degradation: *SPOP*-mutant PCs showed increased PD-L1 expression in a series [75]. Finally, the immune-suppressed PC group showed fewer *SPOP* mutations than the immune-activated and non-immune class in another study (5.6%, 13.5%, and 13.2%, respectively, *p* < 0.001) [21]. 

Systematic literature reviews and metanalyses (SLRs) conducted according to the PRISMA guidelines (including an evidence-based minimum set of items for reporting) (http://www.prisma-statement.org/, accessed on 8 May 2021) are increasingly important in health care, keeping medical doctors up to date, and also representing the background for developing clinical guidelines/trials, as well as the justification for financial supports of research projects. Usually conducted by multidisciplinary teams, SLRs performed according to these guidelines could be applicable in various topics/contexts, improving the research quality not only of pure meta-analyses but also of SLRs applied to case report/series [215,216,217,218,219,220,221,222,223,224,225,226,227,228,229,230,231,232,233,234,235,236,237,238,239,240,241,242,243,244,245,246,247,248,249,250,251,252,253,254,255,256,257,258,259]. To better present the data of our SLR, we have split the discussion of our results into different articles, highlighting relevant subtopics: PD-L1 IHC expression in PC with discussion of pre-analytical and interpretation variables; correlations of PD-L1 expression with clinic–pathological features in PC patients; PD-L1 intracellular signaling pathways in PC cells and regulation of the tumor microenvironment; pre-clinical models (cell lines, mouse models) and experimental treatments affecting PD-L1 expression in PC cells; genetic and epigenetic regulation of PD-L1; PD-L1 expression in liquid biopsies, etc. [260,261,262,263,264,265].

## 5. Conclusions

The NCCN guidelines allow pembrolizumab in selected MSI-H/dMMR mCRPC patients. Excluding cases with clear selection biases, we found 74/677 (11%) PCs with dMMR/MSI: 8/67 (12%) MSI/dMMR cases resulted in PD-L1 positivity. dMMR-PCs (tested by IHC) included ductal PCs (3%), and acinar PCs (14%), while all the cases tested for MSI by molecular analysis were acinar PCs. dMMR may be associated with worse clinical outcome, but further data are required, also as regards the differences in response to therapy. 

*PTEN* is a tumor-suppressor gene regulating cell cycle, proliferation, and apoptosis via different pathways including the *AKT/mTOR*, which upregulates PD-L1 expression in PC. In total, 13/137 (10%) PTEN- PCs were PD-L1+, while 10/29 (35%) PD-L1+ PCs were PTEN-. In PCs, *SPOP* mutations may increase PD-L1 expression, while the potential correlation of PD-L1 with PTEN/ERG IHC positivity should be further clarified.

*BRCA1/2* loss of function is linked to hereditary PCs and HRR deficiency: PARPi can be administered to *BRCA*-mutated PC patients. In total, 15/39 (39%) PCs harbored *BRCA1/2* aberrations. In a recent trial, somatic mutations in *BRCA1/2* or *ATM* had higher responses rates after pembrolizumab in docetaxel-refractory mCRPCs. However, limited data are available for PD-L1 IHC expression in *BRCA1/2*-mutated PC patients; the relation of PD-L1 expression with the DDR genes status is still unclear. Further research has to verify how the efficacy of PD-1 inhibition in mCRPCs could be related to dMMR/MSI, DDR genes defects, or PD-L1 status. 

## Figures and Tables

**Table 1:** PD-L1 expression and microsatellite instability/mismatch repair system protein status in patients with prostatic carcinoma.

Ref.	PD-L1 IHC Positivity Rate (#)	Stage and Treatment	Clinic–Molecular Correlations
[156]	206/206 (100%)	PEM + ENZ vs. PEM to mCRPC (T2-3 M0-1; no PT)	206/206 (100%) MSI-H (all PD-L1+ and MSI-H as to the inclusion criteria).
[19]	0/33 (0%) (#)	Atz to mCRPC (22 with ≥3 PT lines; 32 prior ENZ; 13 prior sipuleucel-T)	2/16 (12.5%) MSI (1 MSH2 loss, 1 MSH2/MSH6 deletion). It was unclear if another MSI-H case showed MSH6 mutations.
[9]	156/258 (60%)	PEM to metastatic or locally confined CRPCs	0/6 (0%) responders (5/6, 83%: PD-L1+) with MSI; 1 long responder (>2 years) with dMMR (IHC) below the cut-off for MSI-H (NGS).
[23,92]	0/28 (0%) (#)	ENZ + PEM to CRPC (pT1c-3 N0-1 M1)	MSI: 1/3 (33%) responders vs. 0/13 (0%) non-responders.
[29,37]	29/220 (13%)	Short-term ADT (DEG) + PEM + WPC to hormone-sensitive PCs (pT2-3ab)	2/220 (0.9%) MLH1- (all with loss of other MMRs); 6/220 (2.7%) MSH2- (all MSH6-); 37/220 (16.8%) MSH6-; 27/220 (12.3%) PMS2-. ML: ≥1 (50/220, 22.7%), ≥2 (15/220, 6.8%), ≥3 (5/220, 2.3%), 4 (2/220, 0.9%). ≥2 ML correlated to higher rate of PD-L1+ PC cells (17.2% vs. 5.2%, *p* = 0.033) (@). No association of MSI with age, family PC history, GS, stage, recurrence, or PD-L1+ PC cells. Significantly elevated preoperative PSA in dMMR men (not for ≥2 ML). Higher BCR risk for ≥1 ML and PD-L1+ TICs (*p* = 0.045).
[38]	20/91 (22%) a; 1/27 (4%) d/m	50 HR; 41 MPC	4/118 (3%) cases (2 MPC, 1 HR, 1 ductal PC) were dMMR (3 MSH2-/MSH6-, 1 PMS2-): only 1/4 (25%) cases was PD-L1+.
[43]	2/42 (5%) a 1/34 (3%) d (#)	NR	4/73 (5%) dMMR PCs (3/40, 8% acinar: 3 MSH6-/PD-L1-; 1/33, 3% ductal: 1 MSH2-/MSH6-/PD-L1- but PD-L1+ immune cells) (*p* = 0.62). Two ductal PCs were indeterminate for MSH6 or MSH2/MSH6.
[80]	1/34 (3%) d 1/30 (3%) a	4/28 ductal N+ (unknown therapy)	1/34 (3%) ductal PCs was MLH1-/PMS2-, while 0/30 (0%) acinar PCs showed dMMR.
[53]	1/19 (5%)	androgen therapy (10 CSPC, 9 CRPC; N1 or M1)	1/17 (6%) tested cases showed MSI (*MSH2* copy number loss) (PD-L1+ by IHC: weak, 5% cells; low rank for PD-L1: 26).
[61]	9/51 (18%)	variable stage; adjuvant RT (some cases)	10/124 (8.1%) dMMR/MSI mCRPCs. Shorter median OS for dMMR/MSI (uni/multivariate analysis; 3.8 vs. 7.0 years; aHR 4.09; 95% CI, 1.52–10.94; *p* = 0.005) (no differences for GS, PSA, age, and stage). dMMR primary PCs strongly associate with developing CRPC. 5/85 (6%) matched HN and CRPC samples had dMMR primary PCs: 4/5 (80%) mCRPC biopsies were dMMR. Higher likelihood of PD-L1 positivity in dMMR mCRPC (5/10, 50% vs. 4/41, 9.8%) (MELRM, OR 14; 95% CI, 2–84; *p* = 0.005). dMMR/MSI mCRPCs have higher D-TIL levels. Germline (*n* = 1) or non-synonymous somatic (*n* = 6) mutations and biallelic events (*n* = 7) in MMR genes (total *n* = 14) had higher dMMR-associated DNA mutational signature activity and mRNA expression signatures. Higher MSI-NGS scores correlated to dMMR mutational signatures. PCs with dMMR mutational signatures overexpressed immune transcripts (CD200R1, BTLA, PD-L1, PD-L2, ADORA2A, PIK3CG, and TIGIT). 5/10 (50%) dMMR mCRPCs were PD-L1+, as 4/41 (9.8%) pMMR.
[68]	39/508 (8%)	ADT in 57 mCRPC (pT2-4 N0-1)	0/2 primary PD-L1+ PCs were MSI.
[56]	2/5 (40%)	Dur + Ola to mCRPC (prior ENZ −/+ ABT)	1/14 (7%) *PMS2* frameshift indel in a *BRCA2* mutated patient (intact second allele; no hypermutation phenotype).
[77]	21/177 (12%): 18/130 (14%) (HN); 3/44 (7%) (AAPL)	pT2/3b Nx/0/1 (44 AAPL; 130 HN)	1/21 (5%) PD-L1+ PCs was MSH2-/MSH6- (GS 9, 5 + 4, pT3bN1, no prior neoadjuvant treatment; “interface pattern” of PD-L1+, TILs).

a: acinar; AAPL: neoadjuvant abiraterone acetate + prednisone and leuprolide; ABT: abiraterone; ADT: androgen deprivation therapy; aHR: adjusted hazard ratio; Atz: atezolizumab; BCR: biochemical recurrence; CI: confidence interval; CRPC: castration-resistant prostate cancer; CSPC: castration-sensitive prostate cancer; d: ductal; DEG: degarelix; dMMR: deficient MMR; D-TILs: density of tumor-infiltrating lymphocytes; Dur: durvalumab; ENZ: enzalutamide; GS: Gleason score; HN: hormone-naïve; HR: high-risk prostate cancer; IHC: immunohistochemistry; m: mixed acinar/ductal; MELRM: mixed-effects logistic regression model; ML: MMR loss; MMR: mismatch repair system proteins; mCRPC: metastatic castration-resistant prostate cancer; MPC: metastatic prostatic cancer; MSI: microsatellite instability; MSI-H: high MSI; NGS: next-generation sequencing; NR: not reported; Ola: olaparib; OR: odds ratio; OS: overall survival; PC: prostate cancer; PEM: pembrolizumab; pMMR: proficient MMR; PT: prior therapy; Ref: reference number; RT: radiation therapy; TICs: tumor-infiltrating immune cells; TILs: tumor-infiltrating lymphocytes; WPC: whole-prostate cryoablation. Notes: (#): The PD-L1 positivity rate refers to positivity in tumor cells. Petrylak et al. [19] found that 33/35 (94%) cases showed absent or <5% positivity in immune cells. Graff et al. [23] reported that 3/28 (11%) cases resulted PD-L1+ in TILs. Lindh et al. [43] described 10/34 (29%) ductal (1–20% cells) and 6/42 (14%) acinar (1–30% cells) cases with PD-L1+ immune cells. (@): not valid for: ≥1 MMR loss and PD-L1+ PC cells (31.0% vs. 21.5%, *p* = 0.340); ≥1 MMR loss and PD-L1+ immune cells (18.2% vs. 23.5%, *p* = 0.653); ≥2 MMR loss and PD-L1+ immune cells (0% vs. 8%, *p* = 0.135).

**Table 2:** PD-L1 expression and status of *BRCA1/2* and other DNA damage repair genes in patients with prostate cancer.

Ref.	PD-L1+ IHC Rate	*BRCA* and Other Relevant DDR Genes	Clinic-Molecular Correlations
[19]	0/33 (0%)	5/16 (31%) *BRCA2* alterations	2/5 (40%) PCs with *BRCA2* aberrations showed PR to Atz (*).
[9]	156/258 (60%)	(1) 19/153 (12%): *BRCA1/2* or *ATM* mut (2) 10/153 (7%): other HHR genes mut (£) (3) 124/153 (81%): no HHR genes mut	(1) 2/19 (11%) ORR to PEM (§), 4/19 (22%) DCR; 2 (11%) PR, 2 (11%) SD, 1 (5%) non-CR/non-PD, 12 (63%) PD, 2 (11%) PSA response. (2) 0% ORR/DCR, 2 (20%) SD, 5 (50%) PD, 1 (10%) PSA response. (3) 4/124 (3%) ORR (RD: 1.9 - ≥ 16.6 mo), 22/124 (18%) DCR; 2 (2%) CR, 2 (2%) PR, 18 (15%) SD, 7 (6%) non-CR/non-PD, 80 (65%) PD, 4 (3%) PSA response. 4/6 (67%) responders (evaluable genomic data) with MSI showed somatic aberrations in ≥1 of 50 DDR genes (°)
[23,92]	0/28 (0%)	4/16 (25%) DDR gene mut	1/3 (33%) ENZ+PEM responders with MSI, DDR genes mut, ≥1 heterozygous cancer-predisposing *ATM* variant. 0/13 non-responders with MSI ($): 3 with heterozygous mut in DDR pathways (2 cancer-associated).
[53]	1/19 (5%)	5/17 *(29%) BRCA1/2* alterations: *BRCA1* (*n* = 1), *BRCA2* (*n* = 2), or *BRCA1/2* (*n* = 1) mut, or *BRCA2* copy loss (*n* = 1) (@)	No association with RNAseq rank of any gene, CRPC vs. CSPC status, or primary PC vs. metastases, nor between DNA mutational profile, CD3/8 IHC status, RNA-seq CD8, PD-L1 IHC status, or TMB and the expression profile of any genes. No difference in the DNA mutational profile (*p* > 0.05) of CRPC vs. CSPC or primary vs. metastatic PC.
[56]	2/5 (40%)	germline (three frameshift *BRCA2* indels + somatic del of the 2nd allele; one *NBN* mut) or somatic (two homozygous *BRCA2* mut) DDR genes alterations	These six patients were responders to durvalumab + olaparib.

(*): 3/14 (21%) PCs (baseline biopsy): *BRCA2* aberrations; 2 PR: (1) *MSH2/MHS6* del; high TMB (30 mut/Mb); *BRCA2* E49*, *TP53* Y236D, *AR* W742C mut; *MYC* amplification; *TMPRSS2-ERG* fusion; PD-L1- baseline biopsy, PD-L1+ post-treatment biopsy (≥5% immune cells, <5% tumor cells); (2) low TMB, microsatellite-stable, *BRCA2/ATM* (?) mut (lymph node metastasis). (§): response duration: 4.4 vs. ≥21.8 mo (*ATM* vs. *BRCA2* mutations). (£): *BARD1*, *BRIP1*, *CDK12*, *CHEK1*, *CHEK2*, *FANCL*, *PALB2*, *PPP2R2A*, *RAD51C*, *RAD51B*, *RAD51D*, and *RAD54L* genes. (°): Four cases: (1) *ATM* splice site acceptor del, *BRCA2* A1162V sub, *CDK12* G1461Afs* del, *FANCA* sub, *FANCD2* R263H sub, *MLH3* T930Qfs*35 del, and *RAD54L* R511H sub; (2) *TP53* R273P sub; (3) *BRCA2* V1176Gfs*8 insertion; (4) *NBN* Q494P and *TP53* S241F sub. ($): a case with one marker of MSI (polymerase chain reaction, not high MSI). (@): details: (1) *BRCA1/2*, *PTEN, FBXW7*, *GATA3*, *SMO*, *TET2*, *TP53*, *TSC1* mut; *MSH2* copy number loss (cnl), PD-L1+; (2) cnl in *BRCA2*, *FBXW7*, *NF2*, *PIK3R1*, *RB1*, *SMAD4*, and *TET2* genes, *PTEN* mut; (3) *BRCA1* mut, *CCND1* copy number gain, *CDKN2A* cnl, *SMAD4* and *TP53* mut; (4) *BRCA2* and *KIT* mut, *TMPRSS2-ERG* fusion; (5) *BRCA2*, *APC*, *PIK3R1*, *RB1*, *TP53* mut. Atz: atezolizumab; CR: complete response; CRPC: castration-resistant prostate cancer; CSPC: castration-sensitive prostate cancer; del: deletion; DCR: disease control rate; DDR: DNA damage repair; ENZ: enzalutamide; HRR: homologous recombinant repair; IHC: immunohistochemistry; mo: months; MSI: microsatellite instability; mut: mutation; ORR: objective response rate (RECIST v1.1 criteria); PC: prostate cancer; PD: progression of disease; PEM: pembrolizumab; PR: partial response; RD: response duration; Ref.: Reference; SD: stable disease; sub: substitution; TMB: tumor mutation burden.

**Table 3:** PD-L1 expression and *PTEN* status in patients with prostatic carcinoma.

Ref.	PD-L1 IHC Positivity Rate	Results
[19]	0/33 (0%)	1/16 (6%) *PTEN* mutations
[38]	20/91 (22%) a; 1/27 (4%) d/m	No association between PD-L1 positivity (26% HR, 17% MPC, 4% ductal PCs) and PTEN or ERG status
[53]	1/19 (5%)	8/17 (47%) *PTEN* mutations (*n* = 5, 29%) or CNL (*n* = 3, 18%) (*). No association with RNAseq rank of any gene, CRPC vs. CSPC status, primary PC vs. metastases, nor between DNA mutational profile, CD3/8 IHC status, RNA-seq CD8, PD-L1 IHC, or TMB and any gene expression profile.
[77]	21/177 (12%): 18/130 (14%) (hormone-naïve); 3/44 (7%) (AAPL)	PD-L1 expression is independent of PTEN status. PTEN IHC loss: - 85/130 (65%) hormone-naïve PCs: 75/112 (67%) PD-L1-/PTEN-; 10/18 (56%) PD-L1+/PTEN-; 37/112 (33%) PD-L1-/PTEN^normal^; 8/18 (44%) PD-L1+/PTEN^normal^ (*p* = 0.345). - 22/44 (50%) neo-AAPL PCs and 20/44 (46%) matched untreated PC controls (*p* = 0.67). In PCs with nodular pattern of PD-L1 positivity (*n* = 7), the PD-L1^high^ and PD-L1^low^ components showed concordant ERG status (°) but variable PTEN staining.
[86]	7/129 (24%)	Loss of PTEN protein expression (IHC, unclear score) in 47/88 (53%) PCs; only 3/47 (6%) PTEN- cases were PD-L1+. *CD274* gene amplification in 1/675 (0.1%) analyzed PCs (@).
[96]	11/20 (55%)	5/20 (25%) PTEN loss (all PD-L1- PCs). No PD-L1+ PCs with PTEN loss (§).

a: acinar; AAPL: neoadjuvant abiraterone, prednisone and leuprolide; CNL: copy number loss; CRPC: castration-resistant prostate cancer; CSPC: castration-sensitive prostate cancer; d/m: ductal or mixed; IHC: immunohistochemistry; HR: high-risk prostate cancer; MPC: metastatic prostatic cancer; PC: prostate cancer; Ref.: reference; TMB: tumor mutation burden. Note: The clinic–pathologic features of a PC series [86] were not reported, while Martin et al. [96] tested a series of radical prostatectomies treated with leuprolide (*n* = 11). For additional molecular information (unrelated to the topic of our review), please read the original articles. (*): The three cases with *PTEN* CNL also showed: (1) CNL in *NF2*, *PIK3R1*, *RB1*, and *TET2*; *TMPRSS2-ERG* fusion; (2) CNL in *APC*, *PIK3R1*, *SMAD4*, and *TET2*; (3) *PTCH1* mutation; *TMPRSS2-ERG* fusion. The five cases with *PTEN* mutations also showed: (1) *BRCA1/2*, *FBXW7*, *GATA3*, *SMO*, *TET2*, *TP53*, and *TSC1* mutations; *MSH2* CNL; PD-L1 IHC positivity; (2) CNL in *FBXW7*, *BRCA2*, *NF2*, *PIK3R1*, *RB1*, *SMAD4*, and *TET2*; (3) CNL in *NF1* and *TSC1*; (4) CNL in *FBXW7* and *RB1*; *TMPRSS2-ERG* fusion; *TP53* mutation; (5) no other reported mutations. Gene alterations included (not being limited to): *AR* gene copy number gain (*n* = 1); *BRCA1* (*n* = 1), *BRCA2* (*n* = 2) or *BRCA1/2* (*n* = 1) mutation or *BRCA2* CNL (*n* = 1); *TMPRSS2-ERG* fusion (*n* = 7); *SPOP* mutation (*n* = 1); *TP53* mutation (*n* = 6); *RB* CNL (*n* = 3) or mutation (*n* = 1), *MSH2* loss (*n* = 1). (°): ERG+: 16/44 (36%) neo-AAPL PCs and 18/44 (41%) matched untreated PC controls (*p* = 0.5); 61/130 hormone-naïve PCs (five ERG+ PCs resulted PD-L1+, *p* = 0.08). The trend of PD-L1+ PCs toward a lower rate of ERG positivity and higher AR expression. The five PDL1+ PCs in ≥ 25% tumor cells were all ERG−. (@): hybrid capture-based comprehensive genomic profiling. (§): assays: IHC (clone D4.3 XP 9271) (negative for markedly decreased intensity or entirely negative staining across all tumor cells compared to the surrounding benign glands and/or stroma).

## Supplementary Materials

The following supporting information can be downloaded at: https://www.mdpi.com/article/10.3390/biomedicines10020236/s1, Table S1: Detailed results of the studies evaluating PD-L1 expression and microsatellite instability/mismatch repair system proteins status in patients with prostatic carcinoma.
