# Supplementary material for: What Do We Have to Know about PD-L1 Expression in Prostate Cancer? A Systematic Literature Review (Part 6): Correlation of PD-L1 Expression with the Status of Mismatch Repair System, BRCA, PTEN, and Other Genes
Source: Biomedicines. 2022 Jan 22;10(2):236. doi: 10.3390/biomedicines10020236 (PMC8868626; doi:10.3390/biomedicines10020236)
Supplement: Supplementary file 1 [file biomedicines-10-00236-s001.zip › biomedicines-1565205-supplementary.pdf]

biomedicines

MDPI

Systematic review

What Do We Have to Know about PD-L1 Expression in Prostate Cancer? A Systematic Literature Review. Part 6: Correlation of PD-L1 Expression with the Status of Mismatch Repair System, *BRCA*, *PTEN*, and Other Genes

Andrea Palicelli [1,*], Stefania Croci [2], Alessandra Bisagni [1], Eleonora Zanetti [1], Dario De Biase [3], Beatrice Melli [4,5], Francesca Sanguedolce [6], Moira Ragazzi [1], Magda Zanelli [1], Alcides Chaux [7], Sofia Cañete-Portillo [8], Maria Paola Bonasoni [1], Stefano Ascani [9,10], Antonio De Leo [11], Guido Giordano [12], Matteo Landriscina [12], Giuseppe Carrieri [13], Luigi Cormio [13], Jatin Gandhi [14] , Davide Nicoli [15], Enrico Farnetti [15], Simonetta Piana [1], Alessandro Tafuni [1,16] and Martina Bonacini [2]

Table S1. Detailed results of the studies evaluating PD-L1 expression and microsatellite instability/mismatch repair system proteins status in patients with prostatic carcinoma.

Ref.	PD-L1 positivity rate, clone and score (IHC)(#)	Clinic-pathological features	Assays/Results (°)
[156]	206/206 (100%)<br><br>(clone 22C3, CPS ≥ 1)	ST: NR<br><br>GG: variable<br><br>Stage: mCRPCs; T2-3 M0-1<br><br>Treatment: (PEM + ENZ) vs. PEM; no P-T	Assay: NR<br><br>Results: 206/206 (100%) MSI-H (all the cases showed PD-L1 positiviy and MSI-H according to the inclusion criteria).
[19]	0/33 (0%) (#)<br><br>(clone SP142, ≥5% of tumor cells)	ST: NR<br><br>GG: variable<br><br>Stage/Therapy: Atezolizumab to mCRPCs (22 with ≥3 P-T lines; 32 prior ENZ; 13 prior sipuleucel-T)	Assay: mutation analyses (FoundationOne assay T7 baitset, Foundation Medicine Inc.), tumor RNA expression analyses (RNASeq, Fluidigm technologies), WES.<br><br>Results: 2/16 (12.5%) MSI, including<br><br>- 1/14 cases (baseline biopsies) (7%): TMB 18 mutations/Mb, MSI-H, *MSH2* loss (no response to atezolizumab, survival time <6 months) (§)<br>- 1 additional case (PR): *MSH2/MHS6* del; high TMB (30 mut/Mb); *BRCA2* E49*, *TP53* Y236D, *AR* W742C mutations; *MYC* amplification; *TMPRSS2-ERG* fusion, PD-L1 (IHC) (baseline: negative; post-treatment: positive in ≥5% immune cells and <5% of tumor cells)

			- 1 additional case (PR): low TMB, microsatellite-stable, *BRCA2*/*ATM* (?) mutation (lymph node metastasis)
[9]	156/258 (60%)<br>(clone 22C3, CPS ≥ 1)	ST: biopsies<br>GG: variable<br>Stage/Therapy: PEM to metastatic or locally confined CRPCs	Assay: WES, mSINGS assay (°°).<br>Results: 0/6 (0%) responders with evaluable genomic data showed MSI (mSINGS), including 4/6 (67%) cases with somatic aberrations in ≥ 1 of 50 DDR genes (*): 1) *ATM* (chr11_108121426-108121426_A_) splice site acceptor del, *BRCA2* A1162V subs (VUS), *CDK12* G1461Afs* del, *FANCA* subs (chr16_89816312-89816312_T_C) (VUS), *FANCD2* R263H subs (VUS), *MLH3* T930Qfs*35 del, and *RAD54L* R511H subs (VUS); 2) *TP53* R273P subs; 3) *BRCA2* V1176Gfs*8 insertion; 4) *NBN* Q494P subs (VUS) and *TP53* S241F subs. A long responder (>2 years) with dMMR (IHC) below the cut-off for MSI-H by mSINGS, high TMB and high CD3+ intratumor infiltration. A long-responder with amplified *CD274*/*PDCD1LG2* locus.
[23,92]	0/28 (0%) (#)<br>(clones E1L3N, 22C3; NR score)	ST: biopsies<br>GG: 1-5<br>Stage: pT1c-3 N0-1 M1<br>Therapy: ENZ + PEM to CRPCs	Assay: PCR (BAT-25, BAT-26, NR-21, NR-24, MONO-27) and NGS (mSINGS) for MSI; WES, gene expression profiling, Knight Diagnostic Laboratory's GeneTrails Solid Tumor Panel, Personal Genome Diagnostics panel (Baltimore, MA, US).<br>Results: 1/3 (33%) responders with MSI (PCR, mSINGS) and > 4000 mutations (also DRG, ≥ 1 heterozygous cancer-predisposing *ATM* variant) (WES) (§§); 0/13 (0%) non-responders with MSI: 1 with 1 marker of MSI (PCR; not MSI-H by mSINGS), 3 with heterozygous mutations in DRG (2 cancer-associated). PD-L1 was negative when performed in cases also tested for MSI (n = 13). No differences in PD-L1 expression or TMB (n = 10; p = 0.42) between responders and non-responders (gene expression profiling); higher number of neoepitopes among responders (679 vs. 405.5, p = 0.42).
[29,37]	29/220 (13%)<br>(clone 22C3; mo/st ≥ 1% or we ≥10%)	ST: RP (TMA)<br>GG: 1-5<br>Stage: pT2-3ab<br>Therapy: Short-term ADT (DEG) + PEM + WPC to	Assay: IHC (clones G168–15, FE11, BC/44, A16–4) (positive: ≥1%).<br>Resuls: 2/220 (0.9%) MLH1- (all with loss of other MMR); 6/220 (2.7%) MSH2- (all with *MSH6* loss); 37/220 (16.8%) MSH6-; 27/220 (12.3%) PMS2-; ≥ 1 MMR loss 50/220 (22.7%); ≥ 2 MMR loss 15/220 (6.8%); ≥ 3 MMR loss 5/220 (2.3%); 4 MMR loss 2/220 (0.9%). ≥ 2 MMR loss was associated with higher rate of PD-L1+ cancer cells (17.2% vs. 5.2%, p = 0.033) (@). 39 (17.7%) men with clinical recurrence or BCR after RP. No

		hormone-sensitive PCs	association of MSI status with age, family PC history, GS, pT/pN stage, and disease recurrence (ç), nor for PD-L1 positivity in cancer cells and clinical outcomes. Higher BCR-risk for ≥ 1 MMR loss and PD-L1+ TICs (p = 0.045). Significantly elevated preoperative PSA levels in dMMR PCs (not for ≥ 2 MMR loss).
[38]	20/91 (22%) acinar (13/50 HR, 7/41, 17% MPC)<br>1/27 (4%) ductal (**)<br>(NR clone; ≥ 1%)	ST: RP, biopsies<br>GG/Stage/Therapy: 50 HR; 41 MPC	Assay: IHC (NR).<br>Results: 4/118 (3%) cases (2 MPC, 1 HR, 1 ductal PC) were dMMR (3 MSH2-/MSH6-, 1 PMS2-): only 1/4 (25%) cases was PD-L1+.
[43]	2/42 (5%) acinar<br>1/34 (3%) ductal (#)<br>(clone SP263; ≥ 1%)	ST: RP, TURP (TMA)<br>GG: 2-5<br>Stage/Therapy: NR	Assay: IHC (positive if at least some nuclei are stained).<br>Results: 4/73 (5%) dMMR PCs (3/40, 8% acinar: 3 MSH6-/PD-L1-; 1/33, 3% ductal: 1 MSH2-/MSH6-/PD-L1- but PD-L1+ immune cells) (p = 0.62). 2 ductal PCs were indeterminate for MSH6 or MSH2/MSH6.
[80]	1/34 (3%) ductal (>10% ductal)<br>1/30 (3%) acinar<br>(clone E1L3N, ≥1%)	ST: TMA (30 acinar, 6 ductal) + 28 non-TMA<br>GG: NR<br>Stage: 4/28 ductal N+<br>Therapy: NR	Assay: IHC (positivity for MMR+ ≥ 1%).<br>Results: 1/34 (3%) ductal PCs were MLH1-/PMS2- (non-TMA), while 0/30 (0%) acinar PCs showed dMMR.
[53]	1/19 (5%) (***)<br>(clone 22C3;<br>NR score)	ST: RP (11), MTS (8)<br>GG: 2-5<br>Stage: pN1 or pM1<br>Therapy: androgen therapy (9 CRPCs, 10 CSPCs)	Assays: OmniSeq Comprehensive®, FISH (copy number gain/loss), PCR (MSI), DNAseq (TMB).<br>Results: 1/17 tested cases showed MSI (*MSH2* copy number loss), as well as high TMB, *PTEN*, *BRCA1/2*, *FBXW7*, *GATA3*, *SMO*, *TET2*, *TP53*, and *TSC1* mutations: it was the only PD-L1+ case (weak, in 5% cells; low rank for PD-L1: 26). No association with RNAseq rank of any gene, CRPC vs. CSPC status, or primary PC vs. metastases, nor between DNA mutational profile, CD3/8 or PD-L1 IHC status, RNA-seq CD8, or TMB and the expression profile of any genes.

[61]	9/51 (18%) (clone E1L3N; TPS)	ST: RP, biopsies, TURP<br>GG/Stage: variable<br>Therapy: adjuvant RT (some cases)	Assay: IHC for MMR (clones FE11, EP49, ES05, EP51) (positivity: nuclear positivity, regardless of intensity), PCR (Promega MSI Assay v1.2), NGS (targeted on MMR gene coding sequences, dMMR_MUT; or dMMR_MSINGS).<br>Results: 10/124 (8.1%) dMMR/MSI mCRPC patients (biopsies) (more frequent false-positive, discordant results with Promega assay), also having higher mutational loads/MSINGS scores. Shorter median OS for dMMR/MSI cases (vs. non-dMMR/MSI; univariate/multivariate analysis; 3.8 vs. 7.0 years; aHR 4.09; 95% CI, 1.52–10.94; *p* = 0.005), while no differences for GS, PSA, age, and stage. dMMR in primary PC strongly associates with developing dMMR CRPCs. 5/85 (6%) matched HN and CRPC samples had dMMR (IHC) in primary PC: in mCRPC biopsies, 4/5 (80%) were diffusely dMMR (IHC), while 1/5 (20%) was pMMR (IHC). 2/5 HSPC and 1 CRPC showed heterogeneous staining. Higher likelihood of PD-L1 positivity in dMMR mCRPC (5/10, 50% vs. 4/41, 9.8%) (MELRM, OR 14; 95% CI, 2–84; *p* = 0.005). Some dMMR/MSI mCRPC have higher D-TIL levels than pMMR (°°°). Germline mutations (*n* = 1), non-synonymous somatic mutations (n = 6), or biallelic events (*n* = 7) in MMR genes (total *n* = 14) had higher dMMR-associated DNA mutational signature activity and mRNA expression signatures. Higher MSI-NGS scores correlated to dMMR mutational signatures. PCs with dMMR mutational signatures overexpressed a variety of immune transcripts, such as CD200R1, BTLA, PD-L1, PD-L2, ADORA2A, PIK3CG, and TIGIT. 5/10 (50%) dMMR mCRPCs were PD-L1+, as 4/41 (9.8%) pMMR
[68]	39/508 (8%) (clone SP263; ≥ 1%)	ST: RP, biopsies, autopsies (TMA)<br>GG: 1-5<br>Stage: pT2-4 N0-1<br>Therapy: ADT in 57 mCRPCs	Assay: Microsatellite Instability Analysis System v1.2, MSI Multiplex Kit; Promega, Madison, WI; ABI 3130XL Genetic Analyzer, Applied Biosystems, Foster City, CA, US<br>Results: 0/2 (0%) primary PD-L1+ PCs were MSI.
[56]	2/5 (40%) (clone E1L3N; ≥ 1%)	ST/GG: NR<br>Stage: metastatic	Assay: Sequencing panel (500 genes).<br>Results: 1/14 (7%) *PMS2* frameshift indel in a *BRCA2* mutated patient (intact second allele; no hypermutation phenotype).

		Therapy: durvalumab + olaparib to mCRPCs (prior ENZ -/+ ABT)	
[77]	21/177 (12%): 18/130 (14%) (HN); 3/44 (7%) (AAPL) (clones E1L3N, SP142; ≥ 1%, mo/st)	ST: RP GG: 1-5 Stage: pT2/3b Nx/0/1 Therapy: 44 AAPL; 130 HN	Assay: IHC (score: 0 for "no loss", 1 for "complete loss in all tumor cells"). Results: 1/21 (5%) PD-L1+ PCs was MSH2-/MSH6- (GS 9, 5+4, pT3bN1, no prior neoadjuvant treatment; "interface pattern" of PD-L1+, TILs).

AAPL: neoadjuvant abiraterone Acetate + Prednisone and Leuprolide; ABT: abiraterone; ADT: androgen deprivation therapy; aHR: adjusted hazard ratio; BCR: biochemical recurrence; CPS: Combined positive score (number of PD-L1+ cells, including tumor cells, lymphocytes, and macrophages, divided by the total number of tumor cells, x 100); CI: confidence interval; CRPC: castration-resistant prostate cancer; CSPC: castration-sensitive prostate cancer; DDR: DNA damage repair genes; DEG: Degarelix; del: deletion; dMMR: deficient MMR; DRG: DNA repair genes; D-TILs: density of tumor-infiltrating lymphocytes; ENZ: enzalutamide; FISH: Fluorescent in situ hybridization; GG: Grade Group; GS: Gleason score; HN: hormone-naïve; HR: high-risk prostate cancer; HSPC: hormone treatment–naive prostate cancer; IHC: immunohistochemistry; MELRM: mixed-effects logistic regression model; MMR: mismatch repair system proteins; mCRPC: metastatic castration-resistant prostate cancer; mo: moderate; MPC: metastatic prostatic cancer; MSI: microsatellite instability; MSI-H: high MSI; MTS: metastases; NGS: next-generation sequencing; NR: not reported; OR: odds ratio; PC: prostate cancer; PCR: Polymerase Chain Reaction analysis; PEM: pembrolizumab; pMMR: proficient MMR; PR: partial response; P-T: prior therapy; Ref: reference number; RP: radical prostatectomy; RT: radiation therapy; ST: sample type; st: strong; Subs: substitution; TICs: tumor-infiltrating immune cells; TILs: tumor infiltrating lymphocytes; TMA: tissue microarray; TMB: tumor mutation burden; TPS: Tumor proportion score; TURP: transurethral resection of the prostate; VUS: variant of unknown significance; we: weak; WES: whole exome sequencing; WPC: whole-prostate cryoablation. (#): the PD-L1 positivity rate refers to positivity in tumor cells. Petrylak et al. [19] found that 33/35 (94%) cases showed absent or <5% positivity in immune cells. Graff et al. [23] reported that 3/28 (11%) cases resulted PD-L1+ in TILs. Lindh et al. [43] described 10/34 (29%) ductal (1–20% cells) and 6/42 (14%) acinar (1–30% cells) cases with PD-L1+ immune cells. (°): only molecular features of patients with data concerning the MMR/MSI status are presented. Further information are provided in the original articles. (§): Other gene mutations (not limited to): *PTEN* (*n* = 1), *AR* (*n* = 4), *TP53* (*n* = 4), *SPOP* (*n* = 2), *BRCA2* (*n* = 3), *MYC* (*n* = 3), *ATM* (*n* = 4); it was unclear if there was another MSI-H case with *MSH6* mutations. (§§): DNA-repair genes examined included *BRCA2, ATM, FANCA, CHEK2, BRCA1, PALB2, HDAC2, RAD51, MLH3, ERCC3, MRE11, NBN, BARD1, BRIP1, CDK12, RAD51B, RAD51C, RAD51D, RAD54L.* (*): all the 6 patients were PD-L1+, except for case 2. DNA damage repair genes examined by Whole-Exome DNA Sequencing: *ATM, ATR, BAP1, BARD1, BLM, BRAP, BRCA1, BRCA2, BRIP1, CDH1, CDK12, CENPQ, CHEK1, CHEK2, EPCAM1, ERCC1, ERCC2, ERCC3, ERCC4, ERCC6, FAM175A, FAM175B, FANCA, FANCC, FANCD2, FANCE, FANCF, FANCG, FANCI, FANCL, GEN1, HDAC2, MLH1, MLH3, MRE11A, MSH2, MSH6, MUTYH, NBN, PALB2, PIF1, PMS2, RAD51, RAD51B, RAD51C, RAD51D, RAD54L, RDM1, TP53*, and *XRCC2*. (**): pure or mixed; (***): another case had an elevated rank (83) for PD-L1 by RNA-seq: only immune cells were PD-L1+. The mean ranks for PD-L1, PD-L2, and PD-1 by RNA-seq in all PC cases were 25, 27, and 18, respectively. (°°): *BRCA1/2* or *ATM* mutations (*n* = 19, 12%), mutations of other homologous recombination genes (*n* = 10, 7%), no homologous recombination gene mutations (*n* = 124, 81%). (°°°): 5/9 (55%) dMMR/MSI PCs in the upper quartile of TILs-density, and 3/5 (60%) showed >10 mutations (>90th percentile; 113-gene panel). None of pMMR samples in the upper quartile of TILs-density had pathogenic DNA repair defects (targeted NGS panel), while 2 pMMR

cases showed deleterious mutations in other pathways (*PIK3CA E542K*; *JAK1 E1051**). Some tumors without variants in MMR genes and with low MSINGS scores had dMMR-associated DNA mutation signatures (unclear significance). (@): not valid for: ≥ 1 MMR loss and PD-L1+ cancer cells (31.0% vs. 21.5%, $p = 0.340$); ≥ 1 MMR loss and PD-L1+ immune cells (18.2% vs. 23.5%, $p = 0.653$); ≥ 2 MMR loss and PD-L1+ immune cells (0% vs. 8%, $p = 0.135$). (ç): disease recurrence: MLH1, $p = 0.373$; MSH2, $p = 0.348$; MSH6, $p = 0.946$; PMS2, $p = 0.681$; ≥ 1 MMR loss, $p = 0.477$; ≥ 2 MMR loss, $p = 0.486$; ≥ 3 MMR loss, $p = 0.352$.
